# The transplant rejection response involves neutrophil and macrophage adhesion-mediated trogocytosis and is regulated by NFATc3

**DOI:** 10.1038/s41419-024-06457-4

**Published:** 2024-01-19

**Authors:** Siyu Zhao, Yunyi Hu, Bicheng Yang, Lichao Zhang, Meiyining Xu, Kefeng Jiang, Zhun Liu, Mingrou Wu, Yun Huang, Peipei Li, Si-Jia Liang, Xi Sun, Geoff Hide, Zhao-Rong Lun, Zhongdao Wu, Jia Shen

**Affiliations:** 1 Zhongshan School of Medicine, Sun Yat-Sen University, Guangzhou, 510080 Guangdong China; 2 Key Laboratory of Tropical Disease Control (Sun Yat-Sen University), Ministry of Education, Guangzhou, 510080 Guangdong China; 3 Provincial Engineering Technology Research Center for Biological Vector Control, Guangzhou, 510080 Guangdong China; 4 The Andrology Department, The First Affiliated Hospital, Sun Yat-sen University, Guangzhou, 510080 China; 5 Key Laboratory for Stem Cells and Tissue Engineering (Sun Yat-Sen University), Ministry of Education, Guangzhou, 510080 Guangdong China; 6 Department of Pharmacology, Cardiac and Cerebral Vascular Research Center, Sun Yat-sen University, 74 Zhongshan 2 Rd, Guangzhou, 510080 China; 7 Biomedical Research and Innovation Centre, School of Science, Engineering and Environment, University of Salford, Salford, M5 4WT UK; 8 State Key Laboratory of Biocontrol, School of Life Sciences, Sun Yat-Sen University, Guangzhou, 510275 China

**Keywords:** Cell death, Cell adhesion

## Abstract

The anti-foreign tissue (transplant rejection) response, mediated by the immune system, has been the biggest obstacle to successful organ transplantation. There are still many enigmas regarding this process and some aspects of the underlying mechanisms driving the immune response against foreign tissues remain poorly understood. Here, we found that a large number of neutrophils and macrophages were attached to the graft during skin transplantation. Furthermore, both types of cells could autonomously adhere to and damage neonatal rat cardiomyocyte mass (NRCM) in vitro. We have demonstrated that Complement C3 and the receptor CR3 participated in neutrophils/macrophages-mediated adhesion and damage this foreign tissue (NRCM or skin grafts). We have provided direct evidence that the damage to these tissues occurs by a process referred to as trogocytosis, a damage mode that has never previously been reported to directly destroy grafts. We further demonstrated that this process can be regulated by NFAT, in particular, NFATc3. This study not only enriches an understanding of host-donor interaction in transplant rejection, but also provides new avenues for exploring the development of novel immunosuppressive drugs which prevent rejection during transplant therapy.

## Introduction

The innate immune system is an important means for the body to remove foreign tissues (microorganisms, parasites, cancer cells, transplanted organs and tissues). Transplantation rejection, a typical example of an immune response against foreign tissues, is the main factor affecting the long-term survival of transplanted organs. Therefore, elucidating the specific mechanism of transplant rejection is crucial for developing new therapeutic strategies to prevent or treat rejection. After implantation, the innate immune response mediated by innate immune cells, such as neutrophils and macrophages, is the first line of defense against the foreign tissue [1–3]. During rejection, neutrophils are recruited to the graft site and contribute to the inflammatory response by releasing pro-inflammatory mediators [4, 5], such as reactive oxygen species (ROS) [6–8], hydrolytic enzymes, neutrophil elastase (NE) and profibrotic factors [9–12], which further attract other immune cells to the site by the released chemokines [13, 14]. Macrophages, on the other hand, are phagocytic cells that engulf and digest foreign particles, including transplanted tissues. They also produce inflammatory mediators and as antigen presentation cells present antigens to activate the adaptive immune response, particularly T lymphocytes, which is a critical step in graft rejection [13, 15–17]. Once activated, T cells release inflammatory cytokines and directly attack the transplanted tissue, which becomes increasingly difficult to stop the rejection process, ultimately resulting in transplant failure [18]. Current anti-rejection therapy strategies mainly targeting adaptive immunity, especially developing the inhibitors of T-cell, unfortunately have shown limited effect on improving graft survival [19, 20]. Thus, this has evoked considerable renewed attention to the role of the innate immune cells in mediating allograft rejection [12, 19, 21–25]. However, the mechanisms underlying the involvement of innate immune cells in transplant rejection have not been fully elucidated, and the potential developing strategies to improve the success of transplantation are still unclear.

In recent years, some new mechanisms of neutrophils and macrophages have been reported to be involved in inflammation responses to infections and tissues injury, such as trogocytosis. Trogocytosis is a widespread form of cell-to-cell interaction in a number of species [26]. It involves one cell coming into contact with and rapidly “biting” another to obtain intact transmembrane proteins without proteolytic cleavage, including MHC molecules, costimulatory molecules, adhesion molecule receptors, tumor antigens and the antigens of pathogens [27–33]. The cell types involved include T cells, B cells, NK cells, APCs (e.g., dendritic cells, monocytes/macrophages, neutrophils, endothelial cells, fibroblasts, eosinophils, basophils, etc.), tumor cells and pathogen cells (e.g., viruses, bacteria and parasites) [27–33]. Trogocytosis is involved in various physiological processes in many species, such as neuronal remodeling, promoting sperm-egg fusion, removing old fat cells, mediating cell rejection during embryonic development, programmed cell remodeling during the development of parasites and amoeba trogocytosis [33–38]. Trogocytosis has also been reported to be involved in hematopoietic stem cell transplantation rejection and plays a dual role [39, 40]. On the one hand, the transfer of intact MHC-I molecules from donor cells to recipient cells through trogocytosis is beneficial to escape immune surveillance of the recipient and attenuate rejection, thus achieving successful transplantation [39, 40]. On the other hand, the process of presenting the donor MHC to the recipient’s immune system through trogocytosis is considered to be one of the pathways that cause transplant rejection. At present, some studies are using this mechanism to achieve graft survival by exhausting recipient cells that obtain donor MHC molecules after organ transplantation [41]. The existing studies on the role of trogocytosis in transplant rejection mainly focus on information-transfer mediated immune cell tolerance in blood system transplantation. Thus far, it is not known whether trogocytosis is involved in the rejection of parenchymal organs (such as blood vessels, heart, kidney, ect.) or whether recipient cells perform trogocytosis-mediated direct damage to grafts.

In this study, we found that neutrophils and macrophages participate in anti-foreign tissue responses (skin transplantation) through contact-dependent trogocytosis. In this process, complement C3 and receptor CR3 were involved in macrophage and neutrophil-mediated adhesion and damage to foreign tissues. In addition, we also proved that the NFAT inhibitors cyclosporine A (CsA) and tacrolimus (FK506), an effective drug currently used in the clinical treatment of transplantation immune rejection, could effectively inhibit trogocytosis-mediated damage. Using the Myeloid-specific NFATc3 knockout mouse (NFATc3^MKO^) model, we further demonstrated that NFATc3 plays an important role in the regulation of the trogocytosis-mediated anti-foreign tissue response. This study suggests that trogocytosis might be a new immune mechanism against foreign tissue (transplant rejection) in vivo and elucidated the key site of regulation of trogocytosis, which provides a new idea for inhibiting the immune responses against foreign tissue and especially transplant rejection.

## Results

### A large number of neutrophils and macrophages adhere to the surface of the graft during skin transplantation

**Fig. 1:**
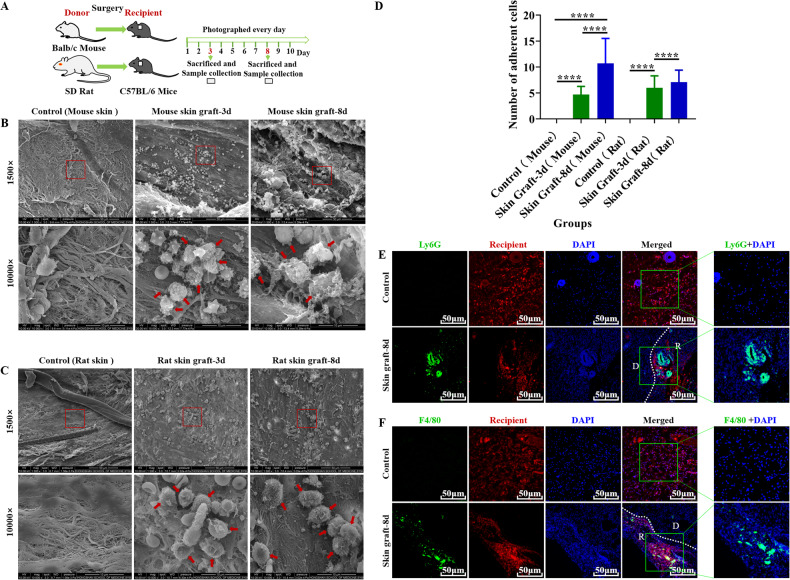
A large number of neutrophils and macrophages adhere to the surface of the graft during skin transplantation. **A** Schematic illustration of skin graft from Balb/C mouse or SD rat to C57BL/6 mice. **B**, **C** Representative scanning electron microscopy (SEM) images showed a large number of leukocyte-like cells (as indicated by the red arrow) attached to the recipient contact surface of the mouse (**B**) or rat (**C**) skin grafts at 3 and 8 days after transplantation. The red arrows indicate adherent cells. Control: recipient mouse skin without undergoing transplantation. Scale bar = 50 µm/10 µm. Magnification = 1500×/10000×. *n* = 5 mice per group. **D** Quantitative analysis of the number of adherent cells. The numerical value represents the number of cells in the field of view at a magnification of 10,000. Data are expressed as the mean ± SD. Statistical analysis was performed using Student’s *t* test. *P < 0.05, **P < 0.01, ***P < 0.001, ****P < 0.0001. *n* = 5 mice per group. **E**, **F** Representative images of immunofluorescence staining of Ly6G (**C**, the marker for neutrophils, green) or F4/80 (**D**, the marker for macrophages, green) in skin grafts tissue. Nuclei are stained with DAPI (blue). D donor, R recipient, Control recipient mouse skin (red) without undergoing transplantation, Skin Graft-8d mouse skin grafts (contained the recipient tissue (red)) 8 days post skin transplantation. The green box contains a partially enlarged view of double labeling image. Scale bar = 50 µm. Magnification = 200×. *n* = 5 mice per group.

During skin graft between Balb/C mouse or SD rat and C57BL/6 mice (Fig. 1A), we observed that a large number of leukocyte-like cells adhered to the recipient contact surface of the grafts at 3 and 8 days after transplantation (Fig. 1B–D). H&E staining showed that many inflammatory cells were infiltrated into the Balb/C mouse or SD rat skin grafts (Supplementary Fig. S1A). To further identify the type of these leukocyte-like cells, immunofluorescence (IF) staining was performed. As shown in Fig. 1E, F, the neutrophil marker Ly6G and macrophage marker F4/80 were significantly expressed in the contact surface between the graft and the recipient tissue, compared with the untreated skin of the recipient mouse (Fig. 1E, F). This result indicated that neutrophils and macrophages may be involved in the adhesion to the recipient contact surface of the grafts.

### Neutrophils and macrophages of C57BL/6 mice can autonomously adhere to and then damage neonatal rat cardiomyocyte mass (NRCM) in vitro

**Fig. 2:**
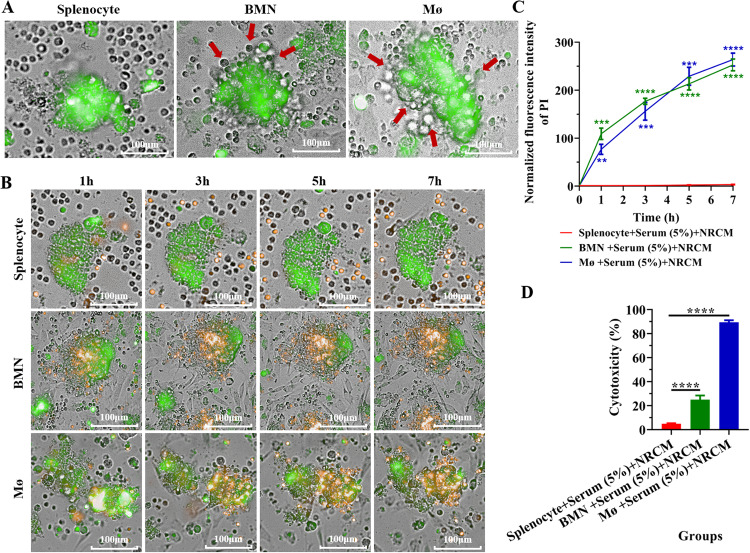
Neutrophils and macrophages of C57BL/6 mice can autonomously adhere to and damage neonatal rat cardiomyocyte mass (NRCM) in vitro. **A** Representative images showing the adhesion of splenocytes, BMN and Mø (bright field) from C57BL/6 mice to the NRCM (labeled with CFDA SE, green) after co-cultured for 7 h. The red arrows indicate adherent cells. No splenocytes were adherent to the NRCM. Scale bar = 100 µm. Magnification = 20×. Three C57BL/6 mice were used and repeated thrice with similar results. **B** Representative images showing the apoptosis of the NRCM (orange) at different times after co-culture with splenocytes, BMN and Mø (bright field) from C57BL/6 mouse. The NRCM were labeled with CFDA SE (green) and the apoptosis of NRCM was detected by propidium iodide staining (orange). Scale bar = 100 µm. Magnification = 20×. **C** Statistical analysis of apoptosis by quantitative PI fluorescence intensity of NRCM at different time points after co-cultured with splenocytes for 7 h, BMN and Mø. Data are expressed as mean ± SD (*n* = 6 per group). Statistical analysis was performed using two-way ANOVA with Dunnett’s multiple comparisons test. *P < 0.05, **P < 0.01, ***P < 0.001, ****P < 0.0001. **D** Determination of NRCM cytotoxicity after co-culture for 6 h with splenocytes, BMN and Mø (bright field) of C57BL/6 mice by non-radioactive cytotoxicity assay (Released LDH in culture supernatants). Data are expressed as the mean ± SD (*n* = 3 per group). Statistical analysis was performed using one-way ANOVA with Dunnett’s multiple comparisons test. *P < 0.05, **P < 0.01, ***P < 0.001, ****P < 0.0001. Similar results were obtained in three repeated experiments.

To explore the effect and consequences of neutrophils and macrophages adhering to the graft-recipient interface, NRCM was introduced as the foreign tissues (grafts) and co-cultured with C57BL/6 mouse bone marrow-derived neutrophils (BMN) or peritoneal macrophages (Mø), which is a mimicking system for the anti-foreign tissue immune response (transplant rejection) in vitro. As shown in Fig. 2A, a large number of BMN and Mø (bright field) were found to adhere to the NRCM (green) after being co-cultured for 6 h supplemented with 5% C57BL/6 mouse serum, while no splenocytes were observed adhered to the NRCM under the same conditions (Fig. 2A). Furthermore, the damage of the NRCM after co-culture with splenocytes, BMN and Mø was measured by propidium iodide (PI) staining (apoptosis, green to orange) and a non-radioactive cytotoxicity assay (Released LDH in culture supernatants). The results showed that BMN and Mø could cause damage to NRCM after adhesion (Fig. 2B–D) and the damage was aggravated with time (Fig. 2B, C). However, this phenomenon was not observed in the co-culture system of splenocytes and NRCM (Fig. 2B, C). These results suggest that C57BL/6 mouse BMN and Mø could cause sustained and significant damage to SD rat derived NRCM after adhesion.

### Complement C3 are required for BMN and Mø-mediated adhesion and damage to the NRCM in vitro

**Fig. 3:**
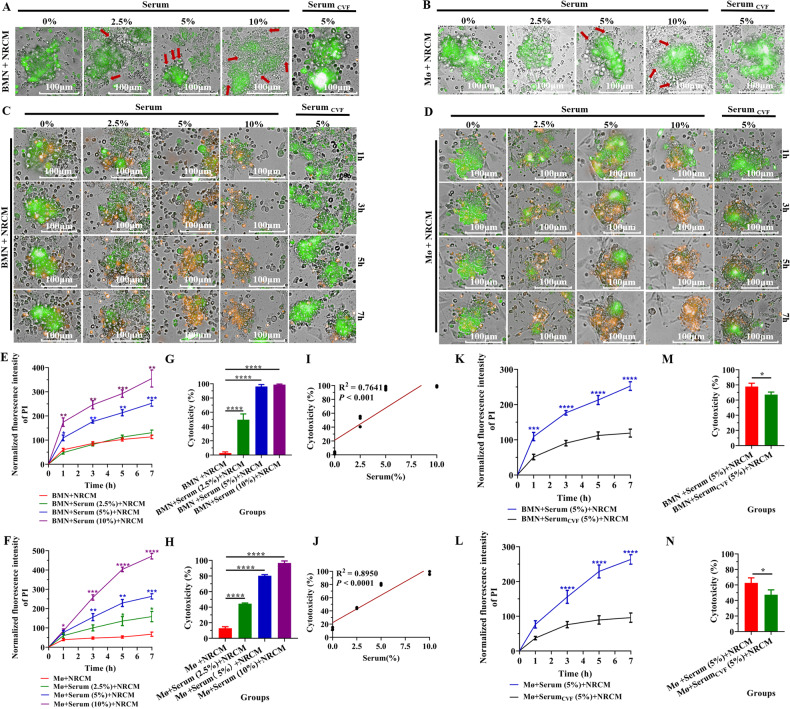
Complement C3 participated in BMN and Mø-mediated adhesion and damage to NRCM in vitro. **A**, **B** Representative micrographs showing the effects of the concentration of normal C57BL/6 mouse serum (%) and C3-inactivated C57BL/6 mouse serum (treated with cobra venom factor (CVF)) in the RPMI-1640 medium on C57BL/6 mouse BMN (**A**, bright field) and Mø (**B**, bright field) attaching to NRCM (green) after co-cultured for 7 h. The red arrows indicate adherent cells. Scale bar = 100 µm. Magnification = 20×. Three C57BL/6 mice were used and repeated thrice with similar results. **C**, **D** Dynamic monitoring of the apoptosis of NRCM (orange) after co-culture with BMN (**C**) and Mø (**D**) (bright field) from C57BL/6 mice at different times for 7 h in the presence of different concentrations of serum and C3-inactivated C57BL/6 mouse serum (treated with CVF) from C57BL/6 mice. NRCM were labeled with CFDA SE (green) and the damage (apoptosis) was detected by propidium iodide staining (orange). Scale bar = 100 µm. Magnification = 20×. Similar results were obtained in three repeated experiments. **E**, **F** Quantitative analysis of the damage (apoptosis) of NRCM mediated by BMN (**E**) or Mø (**F**) by PI fluorescence intensity from **C** or **D**. Data are expressed as the mean ± SD (*n* = 6 per group) and repeated thrice with similar results. Statistical analysis was performed using two-way ANOVA with Dunnett’s multiple comparisons test. *P < 0.05, **P < 0.01, ***P < 0.001, ****P < 0.0001. **G**, **H** Determination of NRCM cytotoxicity induced by C57BL/6 mouse BMN (**G**) and Mø (**H**) after co-cultured for 6 h in the presence of different concentrations of C57BL/6 mouse serum by non-radioactive cytotoxicity assay (Released LDH in culture supernatants). Data are expressed as the mean ± SD (*n* = 3 per group) and repeated thrice with similar results. Statistical analysis was performed using one-way ANOVA with Dunnett’s multiple comparisons test. *P < 0.05, **P < 0.01, ***P < 0.001, ****P < 0.0001. **I**, **J** Correlation analysis of the correlation between the concentration of C57BL/6 mouse serum (%) in the RPMI-1640 medium and the cytotoxicity induced by C57BL/6 mouse BMN (**I**) and Mø (**J**) (non-radioactive cytotoxicity assay) after co-culturing for 6 h in vitro. Correlation analysis was performed using the linear regression model. **K**, **L** Quantitative analysis of PI fluorescence intensity (apoptosis) of NRCM mediated by C57BL/6 mouse BMN (**K**) or Mø (**L**) at different time points in the presence of C3-inactivated serum (treated with CVF) from **C** or **D**. Data are expressed as the mean ± SD (*n* = 6 per group) and repeated thrice with similar results. Statistical analysis was performed using two-way ANOVA with Dunnett’s multiple comparisons test. *P < 0.05, **P < 0.01, ***P < 0.001, ****P < 0.0001. **M**, **N** Determination of NRCM cytotoxicity induced by C57BL/6 mouse BMN (**M**) or Mø (**N**) in the presence of C3-inactivated C57BL/6 mouse serum (treated with CVF) after co-cultured for 6 h by non-radioactive cytotoxicity assay (Released LDH in culture supernatants). Data are expressed as the mean ± SD (*n* = 3 per group) and repeated thrice with similar results. Statistical analysis was performed using Student’s *t* test. *P < 0.05, **P < 0.01, ***P < 0.001, ****P < 0.0001.

We next investigated which factors mediate the adhesion of neutrophils and macrophages to the surrogate foreign tissue, NRCM. Interestingly, we found that both C57BL/6 mouse BMN and Mø-meditated adhesion and the damage to the NRCM were significantly increased in the presence of C57BL/6 mouse serum compared with those in the absence of C57BL/6 mouse serum (0%) (Fig. 3A–D). To further elucidate the relationship between the recipient serum and recipient BMN/Mø-mediated adhesion and damage to the NRCM, the co-culture system of BMN/Mø (bright field) and NRCM (green) was added different concentrations of C57BL/6 mouse serum. As shown in Fig. 3A–F, the adhesion of BMN/Mø to the NRCM (Fig. 3A, B) and the BMN/Mø-mediated NRCM damage were gradually enhanced with the increase of serum concentration in the culture system, which exhibits in a serum dose-dependent manner (Fig. 3C–F). The result of correlation analysis revealed that the cytotoxicity of BMN/Mø to the NRCM was positively correlated with the serum concentration in the culture system (BMN: R^2^ = 0.7641, *P* < 0.001; Mø: R^2^ = 0.8950, *P* < 0.0001) (Fig. 3G–J). Generally, the complement components in serum are closely related to cell adhesion [42]. Subsequently, to elucidate whether complement is a component in serum that influences BMN/Mø cytotoxicity to the NRCM, complement C3 in the serum was depleted by treatment of cobra venom factor (CVF), a functional analog of C3b which can effectively deplete serum C3 within hours [43]. Interestingly, the results showed that the adhesion of BMN and Mø to the NRCM was reduced (right column of Fig. 3A, B) and the damage of NRCM was also significantly decreased (Fig. 3C, D, K–N) at different time points after depletion of complement C3 in serum. Therefore, these data demonstrated that mouse-derived BMN/Mø-mediated adhesion and damage to NRCM required complement C3 of mouse serum.

### CR3 participated in BMN and Mø-mediated adhesion and damage to the foreign tissues (NRCM or skin grafts) in vitro and in vivo

**Fig. 4:**
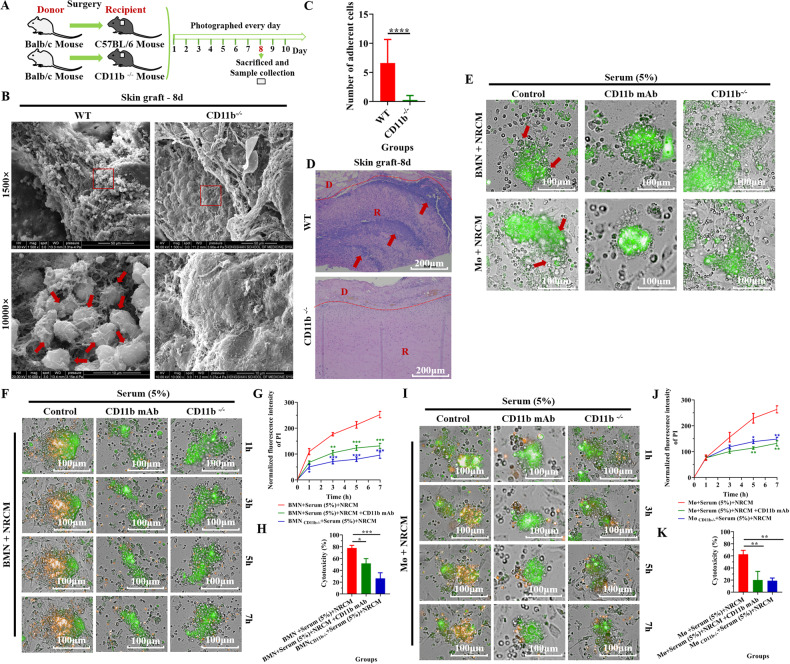
CR3(CD11b) participated in BMN and Mø-mediated adhesion and damage to the foreign tissues (NRCM or skin grafts) in vitro and in vivo. **A** Schematic illustration of skin graft from Balb/C mouse to wild-type C57BL/6 or CD11b^−/−^ mice. **B** Representative SEM images showed cell adhesion (as indicated by the red arrow) to the recipient contact surface of the grafts in wild-type mice and CD11b^−/−^ mice as recipients at 8 days post-transplantation. Scale bar = 50 µm/10 µm. Magnification = 1500×/10000×. *n* = 5 mice per group. **C** Quantitative analysis of the number of adherent cells. The numerical value represents the number of cells in the field of view at a magnification of 10,000. Data are expressed as the mean ± SD. Statistical analysis was performed using Student’s *t* test. *P < 0.05, **P < 0.01, ***P < 0.001, ****P < 0.0001. *n* = 5 mice per group. **D** Representative H&E staining images of the Balb/c mouse skin grafts in wild-type C57BL/6 mice and CD11b^−/−^ mice as recipients at 8 days post-transplantation. Arrows indicate the aggregation of inflammatory cells. D donor, R recipient. Scale bar = 200 µm. Magnification = 100×. *n* = 5 mice per group. **E** Representative images showing the adhesion of BMNs and Mø (bright field) from C57BL/6 mice to NRCM (green) after co-cultured for 7 h when blocking CD11b function with CD11b mAb or knocking out the gene *Itgam* (CD11b^−/−^). The red arrows indicate adherent cells. Scale bar = 100 µm. Magnification = 20×. Three C57BL/6 mice were used and repeated thrice with similar results. **F** Representative images showing the apoptosis of NRCM (orange) at different times for 7 h after co-culture with BMNs (bright field) from C57BL/6 mice when blocking CD11b function with CD11b mAb or CD11b^−/−^ BMNs. NRCM were labeled with CFDA SE (green) and the apoptosis of NRCM was detected by propidium iodide staining (orange). Scale bar = 100 µm. Magnification = 20×. **G** Quantitative analysis of PI fluorescence intensity (apoptosis) of NRCM from **D**. Data is expressed as the mean ± SD (*n* = 6 per group) and repeated twice with similar results. Statistical analysis was performed using two-way ANOVA with Dunnett’s multiple comparisons test. *P < 0.05, **P < 0.01, ***P < 0.001, ****P < 0.0001. **H** Determination of NRCM cytotoxicity induced by C57BL/6 mouse BMN when blocking CD11b function with CD11b mAb or CD11b^−/−^ BMNs after co-cultured for 6 h by non-radioactive cytotoxicity assay (Released LDH in culture supernatants). Data are expressed as the mean ± SD (*n* = 3 per group) and repeated twice with similar results. Statistical analysis was performed using one-way ANOVA with Dunnett’s multiple comparisons test. *P < 0.05, **P < 0.01, ***P < 0.001, ****P < 0.0001. **I**–**K** Determination of the damage to NRCM induced by C57BL/6 mouse Mø when blocking CD11b function with CD11b mAb or CD11b^−/−^ Mø after co-cultured for 7 h by propidium iodide staining (orange) (**I**, **J**) and after co-cultured for 6 h by non-radioactive cytotoxicity assay (**K**). Data are expressed as the mean ± SD (*n* = at least 3 per group) and repeated twice with similar results. Statistical analysis was performed using one-way ANOVA with Dunnett’s multiple comparisons test. *P < 0.05, **P < 0.01, ***P < 0.001, ****P < 0.0001.

We further explore which adhesion molecule mediates the adhesion and damage from mouse BMN/Mø to NRCM. As showed in Supplementary Fig. S2A, compared with the control recipient mouse skin (without undergoing transplantation), the marker for CD11b (green), as a specific subunit of complement receptor 3 (CR3, CD11b/CD18), was expressed at the interface between the graft and the recipient tissue. However, the expression of CD11c, the subunit of complement receptor 4 (CR4, CD11c/CD18) and intercellular adhesion molecule (ICAM1) was not observed at the interface between grafts and recipient tissues (Supplementary Fig. S2B, C). These results suggest that CD11b (CR3) might be an important adhesion molecule participating in the process of BMN/Mø adhesion to foreign tissues. Furthermore, skin grafting between normal Balb/c mouse (donor) and CD11b^−/−^ C57BL/6 mouse (recipient) was performed to investigate the role of CR3 in mediating BMN/Mø adhesion (Fig. 4A). Compared with the control group (wild-type mice as recipients), knockout of CD11b in recipients led to impaired leukocytes adherence to the surface of grafts (Fig. 4B, C) and a decrease in the expression of Ly6G and F4/80 at the interface between the graft and the recipient tissue (Supplementary Fig. S2D, E). In addition, H&E staining showed that many inflammatory cells were infiltrated into the grafts in the wild-type group, whereas the CD11b^−/−^ group showed fewer inflammatory cells infiltrated (Fig. 4D). Similarly, when the function of CR3 in BMN/Mø was blocked with anti-CD11b mAb or with CD11b^−/−^ models in vitro, BMN/Mø-mediated adhesion and damage to NRCM was significantly reduced, compared with the WT BMN/Mø (Fig. 4E–K). Thus, these results demonstrated the receptor CR3 participated in BMN/Mø-mediated adhesion and damage to the foreign tissues (skin grafts or NRCM).

### Trogocytosis participates in BMN/Mø adhesion mediated the damage to the foreign tissues (NRCM or skin grafts)

**Fig. 5:**
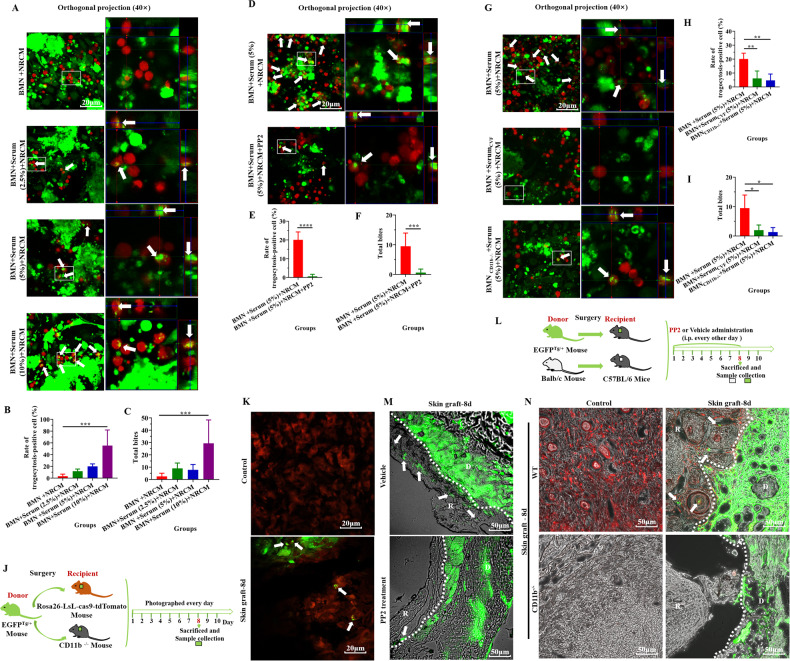
Trogocytosis participates in BMN/Mø adhesion-mediated damage to the foreign tissues (NRCM or skin grafts). **A** Representative images showing BMN (Red) of C57BL/6 mice trogocytosing the NRCM (green) after co-cultured for 12 h in the presence of different concentrations of serum in vitro. Orthogonal views of LSM images displayed BMN trogocytosing the NRCM at different concentrations of serum. The white box contains a partially enlarged view. The white arrows indicate trogocytosing cells. Scale bar = 20 µm. Magnification = 400×. Three C57BL/6 mice were used and repeated thrice with similar results. **B**, **C** Quantitative analysis of trogocytosis-positive BMN (**B**) and total bites of the BMN (**C**) from a. Data are expressed as the mean ± SD (*n* = at least 3 per group) and repeated twice with similar results. Statistical analysis was performed using one-way ANOVA with Dunnett’s multiple comparisons test. *P < 0.05, **P < 0.01, ***P < 0.001, ****P < 0.0001. **D** Representative images showing BMN (red) from C57BL/6 mice trogocytosing the NRCM (green) after co-cultured for 12 h in the presence of PP2 (an inhibitor of trogocytosis) in vitro. The white box contains a partially enlarged view. The white arrows indicate BMN with positive trogocytosis. Scale bar = 20 µm. Magnification = 400×. Three C57BL/6 mice were used and repeated thrice with similar results. **E**, **F** Quantitative analysis of trogocytosis-positive BMN (**E**) and total bites of BMN (**F**) from **D**. Data are expressed as the mean ± SD (*n* = at least 3 per group) and repeated twice with similar results. Statistical analysis was performed using Student’s *t* test. *P < 0.05, **P < 0.01, ***P < 0.001, ****P < 0.0001. Scale bar = 20 µm. Magnification = 400×. **G** Representative images showing BMN (red) from C57BL/6 mice trogocytosing the NRCM (green) after co-cultured for 12 h in the presence of C3-inactivated C57BL/6 mouse serum with CVF and blocking CD11b function (CD11b^−/−^) in vitro. Orthogonal views of LSM images displayed BMN/Mø trogocytosing NRCM at different concentrations of serum, with CVF-treated C57BL/6 mouse serum, or with the knockout of CD11b of BMN of C57BL/6 mice. The white box contains a partially enlarged view. The white arrows indicate trogocytosing cells. Scale bar = 20 µm. Magnification = 400×. Three C57BL/6 mice were used and repeated thrice with similar results. **H**, **I** Trogocytosis-positive rate (**H**) and total bites (**I**) of BMN based on LSM images of each experimental group (CVF-treated serum or knockout of CD11b of BMN from C57BL/6 mouse). Data are expressed as the mean ± SD (*n* = at least 3 per group). Statistical analysis was performed using one-way ANOVA with Dunnett’s multiple comparisons test. *P < 0.05, **P < 0.01, ***P < 0.001, ****P < 0.0001. Similar results were obtained in at least three repeat experiments. **J** Schematic illustration of skin graft from EGFP^Tg/+^ mouse to Rosa26-LsL-cas9-tdTomato Mouse or CD11b^−/−^ mice. **K** Representative LSM images showed the recipient cells (Red) trogocytosing the donor skin material (GFP, green) during skin transplantation in vivo. Control: recipient mouse skin (red) without undergoing transplantation; Skin Graft-8d: green skin grafts (contained the recipient tissue (red)) 8-days post skin transplantation. The white arrows indicate where the cell has “bitten” the donor skin material (trogocytosis). Scale bar = 20 µm. Magnification = 400×. *n* = 5 mice per group. **L** Schematic illustration of PP2 administration after skin graft from EGFPTg/+ mouse or Balb/c mouse into wild-type C57BL/6 mice. **M** Representative images showed trogocytosis on the interface between donor skin (green; D) and recipient skin (TL-PH; R) in control mice (vehicle) and PP2-treated recipient mice at 8 days post-transplantation. The white arrows indicate where the cell has “bitten” the donor skin material (trogocytosis). Scale bar = 50 µm. Magnification = 200×. *n* = 5 mice per group. **N** Representative images showing trogocytosis on the interface between donor skin (green; D) and recipient skin (R) in wild-type mice (red) and CD11b^−/−^ mice (TL-PH) as recipients at 8 days post-transplantation. The white arrows indicate trogocytosing cells. Scale bar = 50 µm. Magnification = 200×. *n* = 5 mice per group.

The above results indicate that the BMN/Mø adhesion-mediated damage to the foreign tissues (NRCM or skin grafts) is highly contact-dependent. This killing method is very similar to the way that neutrophils kill antibody-opsonized tumor cells [44] and the unicellular parasite *Trichomonas vaginalis* in vitro [45], namely trogocytosis, a mechanism which has never been reported in destroying grafts. To explore whether trogocytosis is involved in the damage to the NRCM by BMN/Mø, the mouse BMN/Mø and NRCM were respectively labeled with red and green fluorescence and then trogocytosis was detected after co-cultured for 12 h. As previously described [45], the BMN/Mø (red) containing NRCM material (green) were defined as trogocytosis-positive cells (as shown by the white arrow in Fig. 5A and Supplementary Fig. S3A), the rate of trogocytosis-positive cells (the proportion of gnawing positive cells in the total cells) and the total bites (total number of bites in a single field) were considered the quantitative indicators of trogocytosis intensity. As shown in Fig. 5A and Supplementary Fig. S3A, after the addition of C57BL/6 mouse serum, trogocytosis-positive cells were observed and the trogocytosis intensity of BMN/Mø increased with the increase of serum concentration (Fig. 5B, C; Supplementary Fig. S3B, C). In addition, correlation analysis showed that the trogocytosis intensity of BMN/Mø was positively correlated with serum concentration (Supplementary Fig. S3D–G). However, this trogocytosis of BMN/Mø was able to be inhibited by a trogocytosis inhibitor PP2 [46] (Fig. 5D–F, Supplementary Fig. S4A–C), which led to significantly decreased apoptosis of NRCM (orange) indicated by PI intensity (Supplementary Fig. S4D–F, H) and the damage to the NRCM detected by the non-radioactive cytotoxicity assay (Supplementary Fig. S4G, I). Moreover, when deleting the function of C3 (with CVF-treated mouse serum) or CD11b (with CD11b^−/−^ BMN/Mø) in the co-culture system, we found that the number of trogocytosis-positive cells was significantly decreased or even disappeared (Fig. 5G; Supplementary Fig. S3H) and the trogocytosis intensity of BMN/Mø was significantly reduced (Fig. 5H, I; Supplementary Fig. S3I, J). These results suggest that trogocytosis is involved in the damage to the foreign tissue (NRCM) by BMN/Mø. To capture the evidence of trogocytosis of the host cell in vivo, we transplanted EGFP^Tg/+^ mouse skin (green) into the nape of tdToamto mouse (red) or Balb/c mouse and the trogocytosis of the recipient on the graft was captured (Fig. 5J). As shown in Fig. 5K, we observed the recipient cells (red) contain the graft material (green) (trogocytosis, white arrows). Furthermore, when the inhibitor of trogocytosis PP2 was administered to the skin transplant model (Fig. 5L), the adhesion of recipient cells to grafts (Supplementary Fig. S4J, K), inflammatory cell infiltration (Supplementary Fig. S4L) and the diffusion of graft material (green) into the recipient tissue (Fig. 5M) were significantly inhibited. Additionally, compared the diffusion of green material of the graft into red recipient tissue in WT mice, we did not observe the recipient tissue containing the graft material (green) in CD11b^−/−^ mouse (Fig. 5J, N). Therefore, these results indicate that trogocytosis may be one of the means by which the BMN/Mø damage the NRCM and, therefore, the mechanisms of transplantation rejection.

### NFATc3 regulated BMN/Mø-mediated trogocytosis in the immune response to foreign tissues (NRCM or skin grafts)

**Fig. 6:**
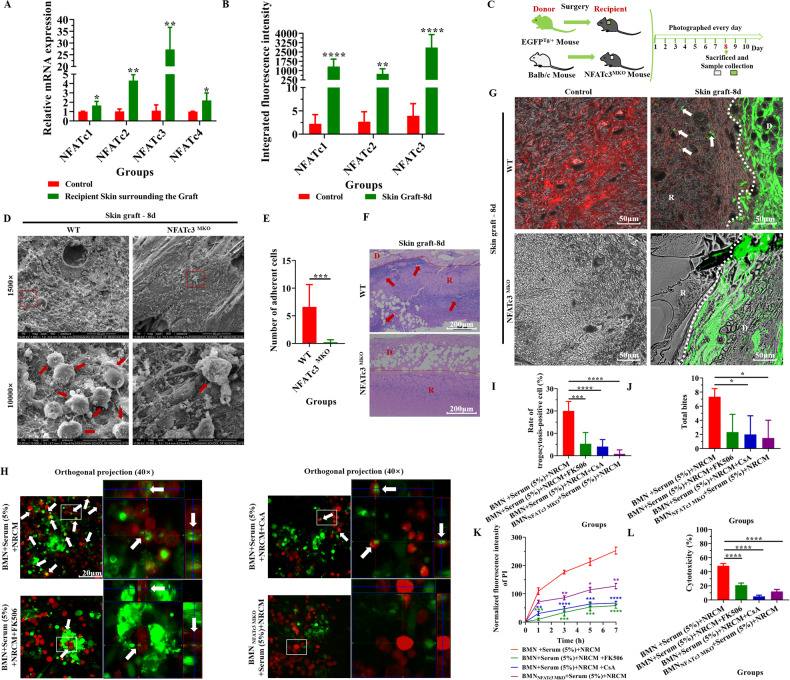
NFATc3 regulated BMN/Mø-mediated trogocytosis in the immune response against foreign tissues (NRCM or skin grafts). **A** Relative mRNA levels of NFATc1-NFATc4 in the normal recipient skin (control) and recipient skin surrounding the graft. Data are expressed as the mean ± SD (*n* = at least 3 per group). mRNA expression is relative to GAPDH. Statistical analysis was performed using Student’s *t* test. *P < 0.05, **P < 0.01, ***P < 0.001, ****P < 0.0001. **B** Quantitative analysis of the expression of NFATc1, NFATc2 and NFATc3 by integrated fluorescence intensity from Supplementary Fig. S5A–C. Data are expressed as the mean ± SD (*n* = at least 5 per group). Statistical analysis was performed using Student’s *t* test. *P < 0.05, **P < 0.01, ***P < 0.001, ****P < 0.0001. **C** Schematic illustration of skin graft from EGFP^Tg/+^ mouse or Balb/c mouse to NFATc3 ^MKO^ mice. **D** Representative SEM images showed cell adhesion (as indicated by the red arrow) to the recipient contact surface of the grafts in wild-type mice and NFATc3^MKO^ mice as recipients at 8 days post-transplantation. Scale bar = 50 µm/10 µm. Magnification = 1500×/10000×. *n* = 5 mice per group. **E** Quantitative analysis of the number of adherent cells. The numerical value represents the number of cells in the field of view at a magnification of 10,000. Data are expressed as the mean ± SD. Statistical analysis was performed using Student’s *t* test. *P < 0.05, **P < 0.01, ***P < 0.001, ****P < 0.0001. *n* = 5 mice per group. **F** Representative H&E staining images of Balb/c mouse skin grafts in wild-type C57BL/6 mice and NFATc3^MKO^ mice as recipients at 8 days post-transplantation. Arrows indicate the aggregation of inflammatory cells. D donor, R recipient. Scale bar = 200 µm. Magnification = 100×. *n* = 5 mice per group. **G** Representative images showed trogocytosis on the interface between donor skin (green; D) and recipient skin (R) in wild-type mice (red) and NFATc3^MKO^ mice (TL-PH) as recipients at 8 days post-transplantation. The white arrows indicate trogocytosing cells. Scale bar = 50 µm. Magnification = 200×. *n* = 5 mice per group. **H**–**L** Analyzing BMN-mediated trogocytosis (**H**–**J**), the damage to NRCM determined by PI staining (**K**) and LDH concentrations in culture supernatants (**L**) after blocking NFAT activation with FK506, CsA and NFATc3^MKO^ BMN in vitro. The white arrows indicate trogocytosing cells. Scale bar = 20 µm. Magnification = 400×. Data are expressed as the mean ± SD (*n* = at least 3 per group) and repeated twice with similar results. Statistical analysis was performed using one-way ANOVA with Dunnett’s multiple comparisons test. *P < 0.05, **P < 0.01, ***P < 0.001, ****P < 0.0001.

The inhibitors of the nuclear factor of activated T cells (NFAT), cyclosporine A (CsA) and tacrolimus (FK506), are widely used to treat and prevent aGVHD in bone marrow transplantation [47] and to prevent rejection of kidney [48], heart [49] and liver [50] transplants. These inhibitors interfere with the dephosphorylation of NFAT and inhibit NFAT (NFATc1-4) translocating into the nucleus to induce target gene expression [51]. We next investigate whether NFAT is involved in the regulation of the process of trogocytosis during transplant rejection. We first detected the expression of NFAT family (NFATc1-NFATc4) in grafts and recipient skin around the graft. The results showed that the mRNA levels of NFATc1-NFATc4 in recipient skin around the graft were significantly higher than that in controls (Fig. 6A). Similarly, the results of immunofluorescence showed that the expression of NFATc1-NFATc3 (non-phosphorylation, activated form) was significantly increased in recipient skin around the graft compared with controls (Supplementary Fig. S5A–C; Fig. 6B). Interestingly, when the NFAT function was inhibited with FK506 in this skin transplant model (Supplementary Fig. S5D), the adhesion of recipient cells to grafts (Supplementary Fig. S5E, F), inflammatory cell infiltration (Supplementary Fig. S5G) and the diffusion of graft material (green) into the recipient tissue (Fig. S5H) were significantly reduced. Considering that the fold changes in NFATc3 expression were the most significant, the role of NFATc3 in regulating the process of trogocytosis was further investigated with NFATc3^MKO^ mice models (Fig. 6C). As shown in Supplementary Fig. S5I, J and Fig. 6D–G, knockdown of NFATc3 led to a decrease in the expression of Ly6G and F4/80 (Fig. S5I, J), adhesion of recipient cells to grafts (Fig. 6D, E), inflammatory cell infiltration (Fig. 6F) and the diffusion of graft material (green) into the recipient tissue (Fig. 6G). In addition, NFAT inhibitors (CsA and FK506) and NFATc3^MKO^ BMN/Mø were introduced into the system mimicking the immune response to the foreign tissue. As shown in Fig. 6H and Supplementary Fig. S6A, the blocking function of NFAT and NFATc3 in BMN/Mø led to a significant reduction in the number of trogocytosis-positive cells (Fig. 6H and Supplementary Fig. S6A), trogocytosis intensity (Fig. 6I, J; Supplementary Fig. S6B, C), apoptosis (Supplementary Fig. S6D–F; Fig. 6K) and damage to the NRCM (Fig. 6L; Supplementary Fig. S6G). Together, these results indicate that NFATc3 might be crucial in regulating the initiation of trogocytosis during the immune response process involved in dealing with foreign tissue.

## Discussion

Transplantation rejection, a major barrier to the long-term survival of transplanted organs, has traditionally depended on the activation of recipient (host) T cells [18]. Unfortunately, current anti-rejection therapeutic strategies mainly targeting T-cell immunity still present limited efficacy in improving graft survival in clinical practice [19, 20]. In contrast, more and more studies have proved that innate immune cells play a unique role in transplant rejection and the role of neutrophils and macrophages in transplant rejection has gradually been uncovered [12, 13, 19, 21, 22, 25]. However, the mechanism of neutrophil and macrophage-mediated tissue damage is not yet fully understood. It has not been elucidated whether neutrophils and macrophages, as the end effector cells of the immune system, can act to influence the status of the graft by direct contact with the graft.

In this study, we found that large numbers of cells adhering to the graft in the skin transplantation experiment. These adherent cells were initially identified as neutrophils and macrophages. Using an in vitro system mimicking the response against foreign tissue, we demonstrated that neutrophils and macrophages can actively adhere to the surface of foreign tissues and cause continuous damage to them. This indicates that recipient-derived neutrophils/macrophages attached to the foreign tissues might be involved in transplant rejection. Although the adhesion of neutrophils and macrophages to the graft has not been reported, the infiltration of neutrophils and macrophages traditionally have been considered a feature of allotransplant rejection [1, 2]. This indicates that the phenomenon of cell infiltration may be similar to the cells adhering to the graft we have discovered here. Currently, it has been found that the depletion of neutrophils and macrophages has been shown to slow T cell-mediated rejection [52]. For example, antibody-mediated (anti-Ly6G) or genetically determined depletion of neutrophils have been found to delay skin rejection or reduce aGVHD aggressiveness and mortality after allogeneic hematopoietic stem cell transplantation (alloHSCT) [53–55]. In mouse heart allograft, macrophage depletion has been shown to significantly reduce murine cardiac allograft vasculopathy (CAV) [56]. The above reports illustrated that the recipient neutrophils or macrophages play a unique role in graft rejection.

We further investigated which molecules mediate the adhesion of neutrophils and macrophages to foreign tissues. In the following experiments, we found that the serum of the recipient increased mouse BMN/Mø-meditated adhesion and subsequent injury to the neonatal rat cardiomyocyte mass (NRCM) in a dose-dependent manner. It is reported that complement, which is abundant in serum, plays an important role in mediating cell adhesion [42]. Complement activation leads to tumor cell opsonization by C3-derived opsonins (C3b, iC3b and C3dg) and the generation of potent pro-inflammatory mediators (C3a and C5a), which in turn recruit and activate immune cells with phagocytic properties (neutrophils and macrophages) [57]. Consistent with these studies, we found that the depletion of C3 in serum with CVF significantly inhibits mouse BMN/Mø-meditated adhesion and damage to NRCM in vitro, suggesting that complement C3 may play an important role in anti-foreign tissue response by mediating cell adhesion. Indeed, many studies have shown that the activation of complement exacerbated allotransplantation injury in several clinical cases [58–60]. In addition, depletion of complement with CVF in vivo can attenuate transplant rejection in a variety of animal models, including cardiac allotransplantation, pig-to-mouse corneal xenotransplantation and renal allotransplantation engraftment in nonhuman primates [61, 62]. Currently, the existing reports on the mechanism of complement C3 participating in transplant rejection focus mainly on its role in promoting local inflammation and regulating T cell-mediated immune response [63, 64]. Our research provides a new mechanism for complement C3 participating in transplant rejection, namely, through mediating cell adhesion. It is reported that C3-meditated cell adhesion required C3b receptors, widely expressed on the surface of BMN/Mø [65]. Furthermore, CR3 (CD11b/CD18) and CR4 (CD11c/CD18) are generally the most commonly recognized receptors by iC3b [66]. In this study, immunofluorescence staining of skin grafts showed that the recipient tissues around the grafts had high expression of CD11b. Subsequent blocking or knockdown of CD11b led to a reduction in the number of neutrophils and macrophages adhering to the foreign tissues and an attenuation in adhesion-mediated damage to the graft or NRCM in vitro and in vivo. These results provide supporting evidence that C3 and the receptor CR3 (CD11b/CD18) participated in neutrophils/macrophages-mediate adhesion and damage to the foreign tissues (NRCM or skin grafts).

We further investigate how neutrophils and macrophages damage the graft after adhesion. Interestingly, using the mimicking system of transplant rejection, we observed trogocytosis occurring, a phenomenon that has (to our knowledge) never been reported in destroying foreign tissues. Moreover, our results showed that the serum of the recipient could enhance the intensity of trogocytosis and facilitate BMN/Mø killing NRCM in vitro. This is consistent with the results of a previous study on neutrophil-mediated trogocytosis killing of *Trichomonas vaginalis* [45]. Previous studies showed that neutrophils and macrophage-mediated trogocytosis played an essential role in killing tumor cells and parasites by destroying their membrane integrity, which led to the loss or disability of organelles and the death of target cells [26, 44, 45]. Taken alongside previous studies, it is suggested that BMN/Mø adhesion mediated by C3-CR3 could damage NRCM or grafts through trogocytosis. However, whether the mechanism of trogocytosis-mediated damage to grafts during the transplant rejection model is similar to that of trogocytosis-mediated damage to tumor cells and parasites still needs more experimental evidence. Previously, the depletion of neutrophils and macrophages has been shown to slow T cell-mediated rejection in the second week [52]. Therefore, whether trogocytosis of neutrophils and macrophages is a way of antigen presentation which promotes the rejection of the graft by other immune cells (such as activating adaptive immunity) also needs further verification.

At present, the mechanism regulating the process of trogocytosis is poorly understood. In this study, we found that cyclosporine A (CsA) and tacrolimus (FK506), both widely-used clinical immunosuppressive drugs, could inhibit BMN/Mø-meditated adhesion, trogocytosis and damage to NRCM. CsA and FK506 both belong to NFAT inhibitors [67]. We further demonstrated the prominent role of NFATc3 in regulating the process of trogocytosis. It is reported that activated NFAT induced epigenetic upregulation of CCR2 chemokine receptors in dorsal root ganglion (DRG) [68] and CXCL14 in the dorsal horn [69]. In polymicrobial sepsis, the activation of the NFAT pathway led to an increase in the expression of chemokines (CXCL1, CXCL2 and CXCL5) and neutrophil migration and infiltration in the lung [70], whereas FK506 impaired central migration of neutrophils [71]. Jiang reported that NFATc1 directly regulates the transcription of *Itgam*, the gene encoding CD11b [72]. These reports demonstrate that NFAT can regulate the transcription and expression of some chemokines and adhesion molecules and then regulate the migration and adhesion of cells. Moreover, it is reported that CsA inhibits cytoskeleton reorganization [73] which is a key requirement for amoebic trogocytosis [74]. Additionally, it has been reported that cell motility and invasion are blocked after inhibition of the NFAT pathway in breast cancer cells [75]. Therefore, these results indicated that NFAT signaling may play an important role in the regulation of trogocytosis by regulation of related gene transcription (such as chemokines, cytokines, *Itgam*, etc.), actin cytoskeleton rearrangement and cell motility.

**Fig. 7:**
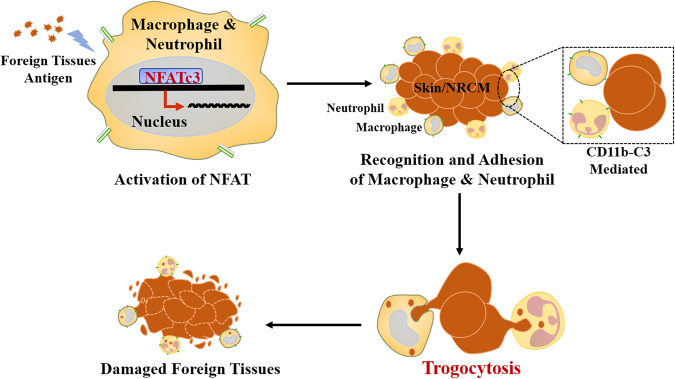
Schematic diagram for the process of macrophage/neutrophil-mediated trogocytosis in graft rejection. Under the regulation of NFATc3, neutrophils and macrophages mediated by CD11b-C3 binding adheres to foreign bodies/grafts and causes damage to them and utilizes the process referred to as trogocytosis.

In conclusion, based on our in vitro model which mimics transplant rejection and the in vivo mouse skin transplantation model, we propose a novel transplant rejection mechanism shown in Fig. 7. This mechanism involves the binding of C3b-CR3 mediated neutrophils- and macrophages- adhesion to foreign tissues and causes damaged to them and utilizes the process referred to as trogocytosis. We further demonstrated that this process can be regulated by a member of the NFAT family, in particular, NFATc3. This study not only enriches an understanding of acceptor-donor interaction in transplant rejection, but also provides potentially new avenues for the development of novel immunosuppressive drugs for use in post-transplantation anti-rejection therapy.

## Materials and methods

### Animals and ethics

8-12-weeks old male Balb/C mice, C57BL/6 mice and 1–3-days old neonatal Sprague–Dawley rats were purchased from Medical Laboratory Animal Center of Guangdong (Guangzhou, China). All genetically engineered mice, including the EGFP^Tg/+^ mice, the Rosa26-LsL-cas9-tdTomato mice, the CD11b^−/−^ mice were purchased from GemPharmatech Co., Ltd (Nanjing, China). The Myeloid-specific NFATc3 knockout mice (*NFATc3*^*MKO*^) were generated by crossing *NFATc3*^*Flox*^ mice with *LysM*-Cre mice as previously described [76]. All animal experiments were approved by the Sun Yat-sen University Committee for Animal Research under license number SYSU-IACUC-2022-B1657 and conformed to the Guidelines for the Care and Use of Laboratory Animals of the National Institute of Health in China.

### Skin transplantation

Full-thickness skin transplantation in mice was performed as described previously [77]. Briefly, the donor mouse (Balb/C mouse, SD Rat, EGFP^Tg/+^ mouse) was anesthetized and the dorsal skin was harvested and cut into 10 mm × 10 mm grafts, which was grafted onto the nape of anesthetized recipient mouse (C57BL/6 mouse, Rosa26-LsL-cas9-tdTomato mouse, CD11b^−/−^ mouse, NFATc3^MKO^ mouse). Post-transplantation mice in the treatment group were injected intraperitoneally with PP2 (2 mg/kg) dissolved in a mixed solvent (50% PEG300 and 50% saline) every other day or FK506 (1 mg/kg) dissolved in the same solvent daily for 8 days, while the control group (vehicle) underwent every two days or daily injection of above solvent for 8 days. The skin grafts were photographed every day until the samples were harvested to assess the survival status of transplants (Supplementary Fig. S7). Grafts were harvested 3 or 8 days later for SEM observation, frozen sections and immunofluorescence staining.

### Scanning Electron Microscopy (SEM)

The grafts were fixed in 2.5% glutaraldehyde for 24 h at 4 °C. The samples were then subjected to a dehydration process that involved 10-minute serial incubation in graded ethanol solution at increasing concentrations of ethanol: 50%, 70%, 85% and 100% (twice for 100%). Excess ethanol was removed by a critical point dryer. Finally, the samples were mounted with double-sided carbon tape onto aluminum cylindrical stubs and plated with gold layers to enhance their electrical conductivity. All samples were imaged by FEI QUANTA 200 SEM at 10 kV or 20 kV.

### Histology and immunofluorescence (IF)

For hematoxylin and eosin (H&E) staining, the tissues of mice were fixed in 4% paraformaldehyde (PFA) overnight at 4 °C, followed by dehydration in 70% ethanol. After the dehydration procedure, tissues were embedded in paraffin, sectioned at a thickness of 8–10 μm and stained with H&E following a standard protocol. Images were acquired using a revolve microscope (Leica DMi1). For immunofluorescence staining, paraffin slides containing sections were incubated twice in xylene for 15 min each, dehydrated twice in anhydrous ethanol for 5 min, followed by dehydrating respectively in gradient ethanol of 85% and 75% ethanol for 5 min each and washed in distilled water. After immersing the slides in EDTA antigen retrieval buffer (pH 6), maintaining at a boiling temperature for 8 min, standing for 8 min, boiling temperature for 7 min again, cooling to room temperature and washing three times with PBS (pH 7.4) for 5 min each, the tissues were incubated in 3% H_2_O_2_ at room temperature for 25 min to block endogenous peroxidase, then washed three times with PBS (pH 7.4) for 5 min each. After eliminating obvious liquid, the objective tissue was marked with a liquid blocker pen and covered with 3% BSA to block non-specific binding for 30 min. Then, they were respectively incubated with rabbit anti-F4/80 (1:500, GB11027, Servicebio), rabbit anti-CD11b (1:500, GB11058, Servicebio), rabbit anti-Ly6G (1:300, GB11229, Servicebio), rabbit anti-CD11c (1:300 GB11059, Servicebio), rabbit anti-ICAM1 (1:500, GB11106, Servicebio), mouse anti-NFATc1 (1:100, sc-7294, Santa Cruz), mouse anti-NFATc2 (1:100, sc-7296, Santa Cruz), mouse anti-NFATc3 (1:100, sc-8405, Santa Cruz) overnight at 4 °C in a wet box and with Cy5-labeled anti-rabbit IgG (1:400, GB27303, Servicebio) or GFP-labeled anti-mouse IgG (1:400, GB25301, Servicebio) at room temperature for 2 h in dark condition. Subsequently, they were incubated with DAPI (Servicebio, China) solution at room temperature for 10 min in a dark place and treated with an anti-fade mounting medium. Images were detected and collected by Leica DMi8 fluorescent microscopy. DAPI glows blue by UV excitation wavelength 330–380 nm and emission wavelength 420 nm; Cy5 glows red by excitation wavelength 648 nm and emission wavelength 662 nm; GFP glows green by excitation wavelength 465–495 nm and emission wavelength 515–555 nm.

### Neonatal Rat Cardiomyocyte (NRCM) isolation and culture

Neonatal rat cardiomyocytes (NRCM) were isolated from Sprague–Dawley rats 1–3 days after birth using a similar protocol described previously [78]. In brief, cardiomyocytes were obtained by digestion of neonatal rat hearts in DMEM/F12 medium (Gibco, Thermo Fisher Scientific, USA) containing 1 mg/ml type II collagenase (Sigma, USA) for 5–6 min each time, 3–4 times in total under sterile conditions. Cardiac fibroblasts were separated by plating on 10 mm culture dishes for 1 h to allow them to attach, leaving the myocytes in the media. Cardiomyocytes were plated on CellCarrier-96 ultra microplates (PerkinElmer, USA) treated with 0.1% gelatin (Sigma, USA) in a medium containing 89% DMEM, 10% FBS and 1% penicillin-streptomycin solution (100 units/mL penicillin G, 100 mg/mL streptomycin) (Gibco, Thermo Fisher Scientific, USA) and incubated for 12–24 h at 37 °C. The next day, NRCM were twice washed gently with PBS and maintained in a suitable complete medium at 37 °C, before use in experiments.

### Isolation of BMN/Mø from C57BL/6 Mice

Bone marrow-derived neutrophils (BMN) of mice were isolated from femurs and tibias of C57BL/6 mice by Percoll (GE Healthcare, USA) density gradient centrifugation as previously described [79]. Marrow cavities of the tibias and femurs of 8-week-old mice were flushed with RPMI1640 complete media. Bone marrow cells were pelleted by centrifugation and suspended in 2 mL of 1×PBS buffer (Gibco, Thermo Fisher Scientific, UK). 2 mL each of the 80% (vol/vol), 65% (vol/vol) and 55% (vol/vol) Percoll in PBS was layered successively into 15 ml centrifuge tube. Finally, cells in 1×PBS buffer were layered on top of the gradient. After centrifugation at 1200 g for 30 min, mature neutrophils at the interface of the 65% and 80% fractions were collected, which were more than 95% viable as determined by trypan blue exclusion. The remaining erythrocytes were lysed with 1×RBC lysis Buffer (Servicebio, Wuhan, China). To isolate peritoneal macrophages, a C57BL/6 mouse was sacrificed and a peritoneal lavage was conducted with 10 mL cold PBS. The lavage fluid was centrifuged and erythrocytes were lysed by the addition of 1×RBC lysis Buffer (Servicebio, Wuhan, China). The erythrocyte-free peritoneal cells were resuspended in RPMI 1640 medium (Gibco, Thermo Fisher Scientific, USA) supplemented with 10% heat-inactivated fetal bovine serum (Gibco, Thermo Fisher Scientific, Australia), 1% penicillin-streptomycin solution and seeded into T25 cell culture flasks and incubated at 37 °C with 5% CO_2_ for 4 h. After 4 h incubation, non-adherent cells were removed by repeated flushing of the flasks with culture medium to obtain pure peritoneal macrophages. For subsequent treatment, both types of cells were centrifuged and the cell pellet was resuspended in 1 mL pre-warmed (37 °C) RPMI 1640 medium supplemented with 10% FBS, 1% penicillin-streptomycin solution. Subsequently, these cells were counted using a hemocytometer and incubated at 37 °C with 5% CO_2_ for later use.

### Labeling BMN/Mø and NRCM

For labeling BMN/Mø, we removed the medium by centrifugation at 500 g for 5 min and the cell pellet was washed with 1×PBS to remove serum and resuspended the cells gently in pre-warmed CellTracker^TM^ Deep Red working solution (8 μM). The cells were incubated for 45 min at 37 °C with 5% CO_2_. The working solution was removed by centrifugation and pre-warmed RPMI 1640 complete medium was added to gently resuspend the cell pellet, which was counted using a hemocytometer and incubated at 37 °C with 5% CO_2_ for later use. The approximate excitation and emission filters for CellTracker^TM^ Deep Red are 630 nm and 660 nm, respectively. The protocol of labeling NRCM by CFDA SE Cell Tracer is similar to that of labeling BMN/Mø, with the main differences in the following three points. Firstly, the working concentration of the CFDA SE Cell Tracer is 2 μM. Secondly, after the removal of the probe, the cells were incubated in RPMI 1640 complete medium for another 30 min to ensure complete modification of the probe and then the cells were washed again. Thirdly, the approximate excitation and emission peaks of this product after hydrolysis are 492 nm and 517 nm, respectively.

### CVF application

The Cobra Venom Factor (CVF) frozen powder (Quidel, USA) purified from *Naja naja*, was dissolved in normal saline to a stock solution at a concentration of 1 mg/mL. According to the instructions, one unit of CVF is equal to 2–6 μg of CVF in general. For in vitro experiments, 8–20 units/mL of serum is adequate to convert nearly all the available C3 to C3 fragments when incubated with neat human serum for 60 to 90 min at 37 °C, which will also convert nearly all the available C5 to C5a and SC5b-9. 80 μL stock solution of CVF was added to 1 mL serum from C57BL/6 mouse and then mixed and incubated for 90 min at 37 °C to prepare mouse serum with complement depletion (CVF-treated C57BL/6 mouse serum) and stored at −20 °C until use.

### BMN/Mø-NRCM coculture

The CFDA SE Cell Tracer-labeled NRCM medium was removed and the cell density of labeled and unlabeled BMN/Mø was adjusted to 5 × 10^4^ cells per well for seeding in 96-well plates containing labeled NRCM. Then, 10 μg/mL propidium iodide (PI, MedChemExpress, USA), the different concentrations of normal C57BL/6 mouse serum, 5% CVF-treated C57BL/6 mouse serum, 60 μg/mL CD11b mAb (NeoBioscience, China), 20 μM PP2 (MedChemExpress, USA), 10 ng/mL FK506 (MedChemExpress, USA), or 50 ng/mL CsA (MedChemExpress, USA) was added to the corresponding experimental wells according to the experimental design. Three repeated wells were set for each experimental group. Subsequently, experimental plates were either placed into the appropriate instrument for the detection or incubated at 37 °C and 5% CO_2_ according to the purpose of the experiment.

### Capturing images of adhesion, trogocytosis and damage

All kinds of microscopes used for image acquisition in this study were obtained from core facilities for medical science (Zhongshan School of Medicine, Sun Yat-sen University, Guangzhou, China). PE Operetta CLS high-content analysis system was used to continuously monitor and record the adhesion and damage (PI: 535 nm/615 nm) of mouse cells to NRCM at 37 °C and 5% CO_2_ for 6 h during co-culture in each well at 20× magnification. Each well was divided into 36 blocks and photographed once an hour on average. Harmony 4.1 software was used to analyze and process the images above. Each of these controls, such as no-cell control and vehicle-only cells control (Supplementary Fig. S8) was performed on each plate being assayed. The average values of PI fluorescence intensity for no-cell control (NRCM only), vehicle-only cells control (BMN, Mø, BMN_CD11b−/−_, Mø_CD11b−/−_, BMN _NFATc3 MKO_ or Mø _NFATc3 MKO_ only) and blank control (medium with equal amount of PI) were subtracted from all PI fluorescence intensity values for experimental total fluorescence intensity of PI. Subsequently, data normalization was performed by dividing the data at each time point by the data at time 0. Images of trogocytosis at different magnifications after incubation for 12 h were collected using ZEN 3.1 Black software connected to a ZEISS LSM 780 confocal microscope. We adjusted the Z-axis of the 3D image for observation, regarded the independent green patches in the red cells as a “bite” and counted the total number of independent green patches in a single field of view as the total number of “bites”. ZEN 3.1 Blue software was used to analyze and process the images above.

### Determination of toxicity of BMN, Mø and splenocytes from C57BL/6 mouse to NRCM

Toxicity of BMN, Mø and splenocytes from C57BL/6 mouse to NRCM was evaluated by measuring lactate dehydrogenase (LDH) activity released in the media after cocultivation for 6 h using the CytoTox96 nonradioactive assay (Promega, USA) and quantitated by measuring wavelength absorbance at 490 nm. Each of these controls, such as no-cell control, vehicle-only cells control and maximum LDH release control, was performed on each plate being assayed. The average absorbance value for the culture medium background was subtracted from all absorbance values for Experimental, Target Cell Spontaneous LDH Release and Effector Cell Spontaneous LDH Release and the following formula was used to calculate percentage cytotoxicity. Cytotoxicity (%) = 100 × (Experimental – Effector Spontaneous – Target Spontaneous)/(Target Maximum – Target Spontaneous). Effector Spontaneous means spontaneous LDH release of splenocytes, BMN, Mø, BMN_CD11b-/-_, Mø_CD11b-/-_, BMN _NFATc3 MKO_ or Mø _NFATc3 MKO_. Target Spontaneous means NRCM spontaneous LDH release.

### Gene expression analysis

Total RNA was extracted from tissue using TRIzol reagents (Invitrogen, USA), according to the manufacturer’s instructions. The obtained RNA was quantified by NanoDrop ND-2000 spectrophotometer (Thermo Scientific, USA). Total RNA was reverse-transcribed into complementary DNA (cDNA) by the Evo M-MLV RT Premix (Accurate Biotechnology, China). The reverse-transcribed RNA was amplified with the appropriate primers listed in Supplementary Table 1. Real-time qPCR analysis was performed using SYBR Green QPCR Master Mix (TaKaRa, Japan) according to the manufacturer’s instructions. The reaction process comprised initial denaturation at 95 °C for 30 s, 95 °C for 5 s, 60 °C amplification for 30 s and 45 cycles of amplification. The *GAPDH* gene was used as an internal control and the fold change was calculated by the formula 2^−ΔΔCt^.

### Statistical analysis

Data were expressed as the mean ± standard deviation (SD). The data for each condition were accumulated from at least three independent experiments. Differences between two groups were evaluated with the two-tailed Student’s *t* test. Multiple comparisons among more than two groups were assessed by performing one-way ANOVA followed by Dunnett’s multiple comparisons test or two-way ANOVA followed by Dunnett’s multiple comparisons test. A value of *P* < 0.05 was considered statistically significant. Correlation analysis was performed using the linear regression model. No statistical methods were used to predetermine the sample size. Mice were randomly allocated to experimental groups. No blinding method was used for injection. There were no animal exclusion criteria. The variance was similar between the groups that were being statistically compared. The statistical analysis of the data was performed using the GraphPad Prism software (version 8.0).

### Supplementary information

Supplementary Materials

Reproducibility checklist

## Data Availability

All data are available in the main text or the supplementary materials. All data, models and materials generated or used during the study are available from the corresponding authors upon reasonable request.
