# Supplementary material for: The transplant rejection response involves neutrophil and macrophage adhesion-mediated trogocytosis and is regulated by NFATc3
Source: Cell Death Dis. 2024 Jan 19;15(1):75. doi: 10.1038/s41419-024-06457-4 (PMC10798984; doi:10.1038/s41419-024-06457-4)
Supplement: Supplementary file 1 — Supplementary Materials [file 41419_2024_6457_MOESM1_ESM.docx]

**
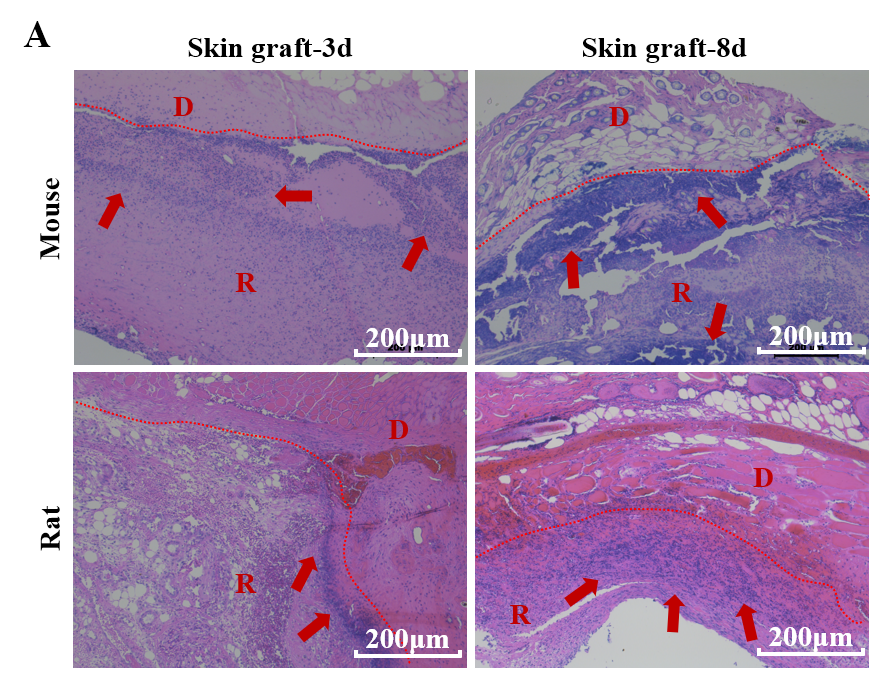
**

**Fig. S1. Inflammatory cells infiltrate skin grafts between Balb/C mouse or SD rat and C57BL/6 mice.**

**A** Representative H&E staining images of the Balb/c mouse or SD rat skin grafts in wild-type C57BL/6 mice as recipients at 3- or 8-days post-transplantation. Arrows indicate the aggregation of inflammatory cells. D: donor; R: recipient. Scale bar = 200µm. Magnification = 100×. n= 5 mice per group.


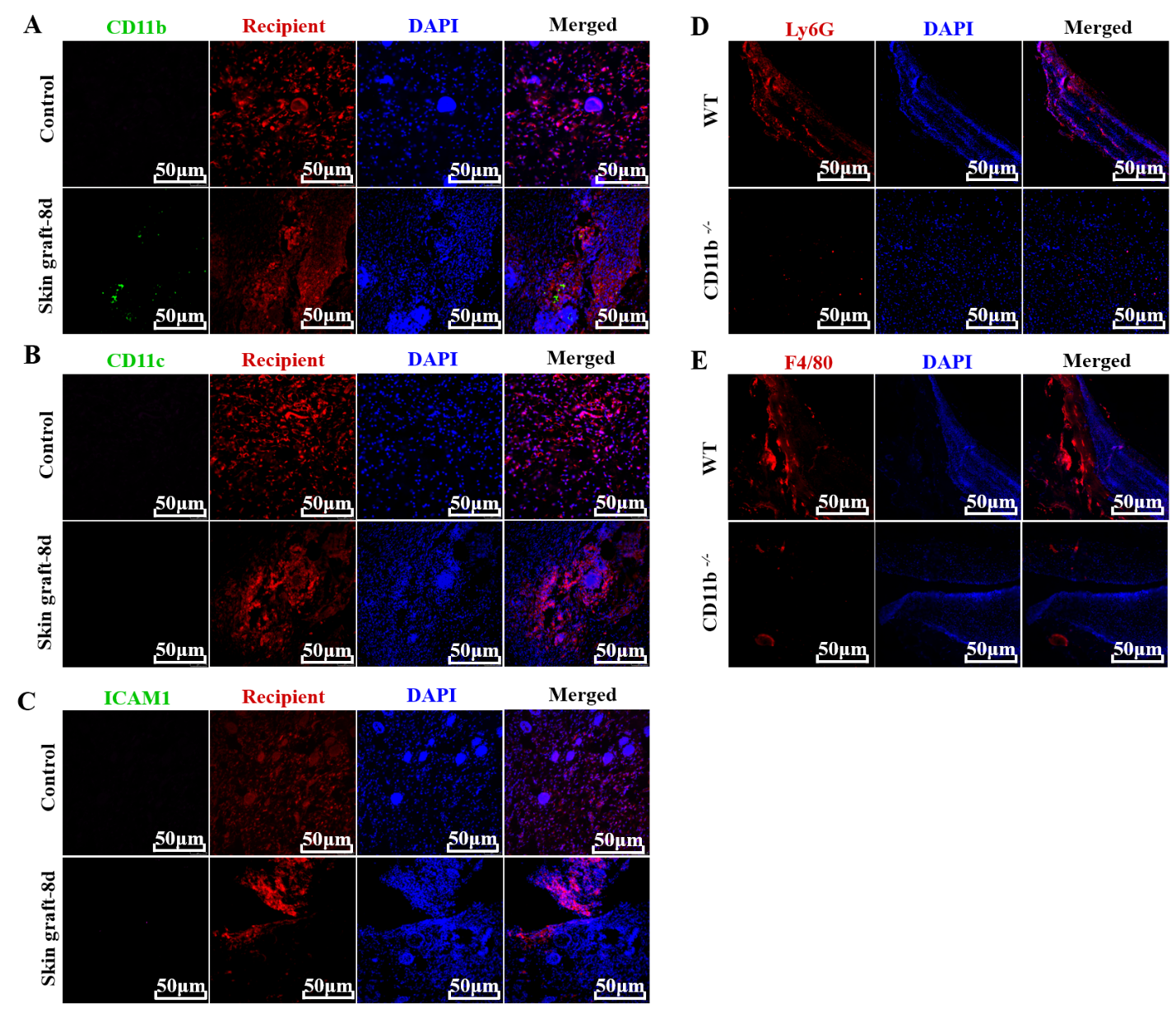


**Fig. S2. Expression of CD11b, CD11c, ICAM1, Ly6G and F4/80 in the recipient tissue attached to skin grafts.**

**A-C** Representative images of immunofluorescence staining of adhesion molecules CD11b (A, green), CD11c (B, green) and ICAM1 (C, green) in 8-day skin graft tissues and normal recipient skin (control). Recipient tissues are displayed in red. Nuclei are stained with DAPI (blue). Control: recipient mouse skin (red) without undergoing transplantation; Skin Graft-8d: skin grafts (contained the recipient tissue (red)) 8-days post skin transplantation. Scale bar = 50µm. Magnification = 200×. n= 5 mice per group. **D-E** Representative images of immunofluorescence staining of Ly6G (D, the marker of neutrophils, red) or F4/80 (E, the marker of macrophages, red) in skin grafts tissue. Nuclei are stained with DAPI (blue). WT: wild-type C57BL/6 mice as transplant recipients; CD11b^-/-^: CD11b^-/-^ mice as transplant recipients. Scale bar = 50µm. Magnification = 200×. n= 5 mice per group.


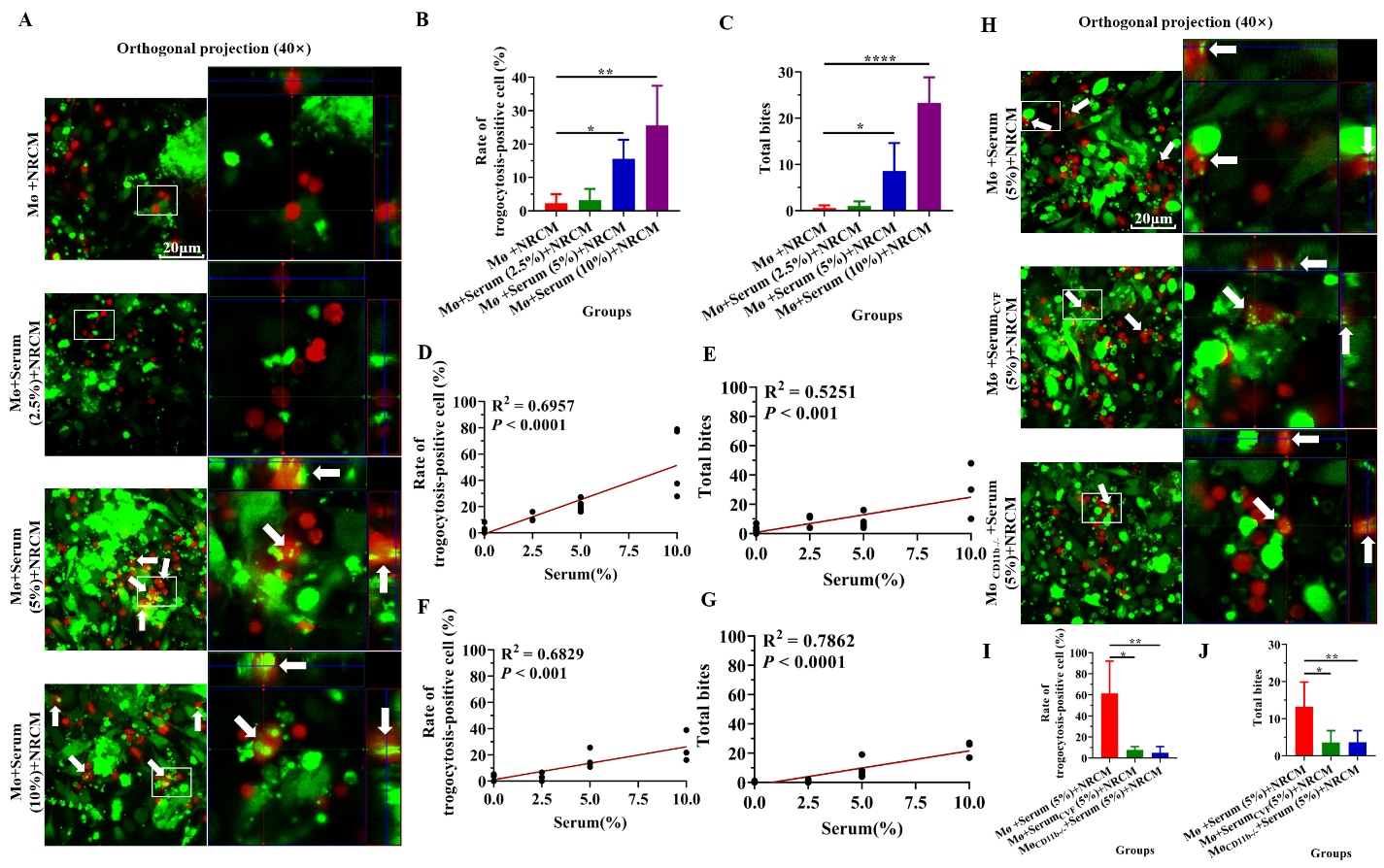


**Fig. S3. Trogocytosis participates in BMN/Mø adhesion mediated the damage to the foreign tissues (NRCM or skin grafts).**

**A** Representative images showing Mø (Red) of C57BL/6 mice trogocytosing the NRCM (green) after co-cultured for 12h in the presence of different concentrations of serum *in vitro*. Orthogonal views of LSM images displayed Mø trogocytosing NRCM at different concentrations of serum. The white box contains a partially enlarged view. The white arrows indicate trogocytosing cells. Scale bar = 20µm. Magnification = 400×. Three C57BL/6 mice were used and repeated thrice with similar results. **B, C** Quantitative analysis of trogocytosis-positive Mø (B) and total bites of Mø (C) from A. Data are expressed as the mean ± SD (n = at least 3 per group) and repeated twice with similar results. Statistical analysis was performed using one-way ANOVA with Dunnett's multiple comparisons test. **P* < 0.05, ***P* < 0.01, ****P* < 0.001, *****P* < 0.0001. **D-G** Correlation analysis of the correlation between the concentration of C57BL/6 mouse serum (%) in the RPMI-1640 medium and the total bites of BMN (D and E) / Mø (F and G) after co-culturing for 6h *in vitro*. Correlation analysis was performed using the linear regression model. **H** Representative images showing Mø (red) of C57BL/6 mice trogocytosing the NRCM (green) after co-cultured for 12h in the presence of C3-inactivated serum with CVF and blocking CD11b function (CD11b^-/-^) *in vitro*. Orthogonal views of LSM images displayed Mø trogocytosing NRCM at different concentrations of serum, with CVF-treated serum, or with the knockout of CD11b of BMN/Mø of C57BL/6 mouse. The white box contains a partially enlarged view. The white arrows indicate trogocytosing cells. Scale bar = 20µm. Magnification = 400×. Three C57BL/6 mice were used and repeated thrice with similar results. **I-J** Trogocytosis-positive rate (I) and total bites (J) of Mø based on LSM images of each experimental group (CVF-treated serum or knockout of CD11b of Mø from C57BL/6 mice). Data are expressed as the mean ± SD (n = at least 3 per group) and repeated twice with similar results. Statistical analysis was performed using one-way ANOVA with Dunnett's multiple comparisons test. **P* < 0.05, ***P* < 0.01, ****P* < 0.001, *****P* < 0.0001.

**
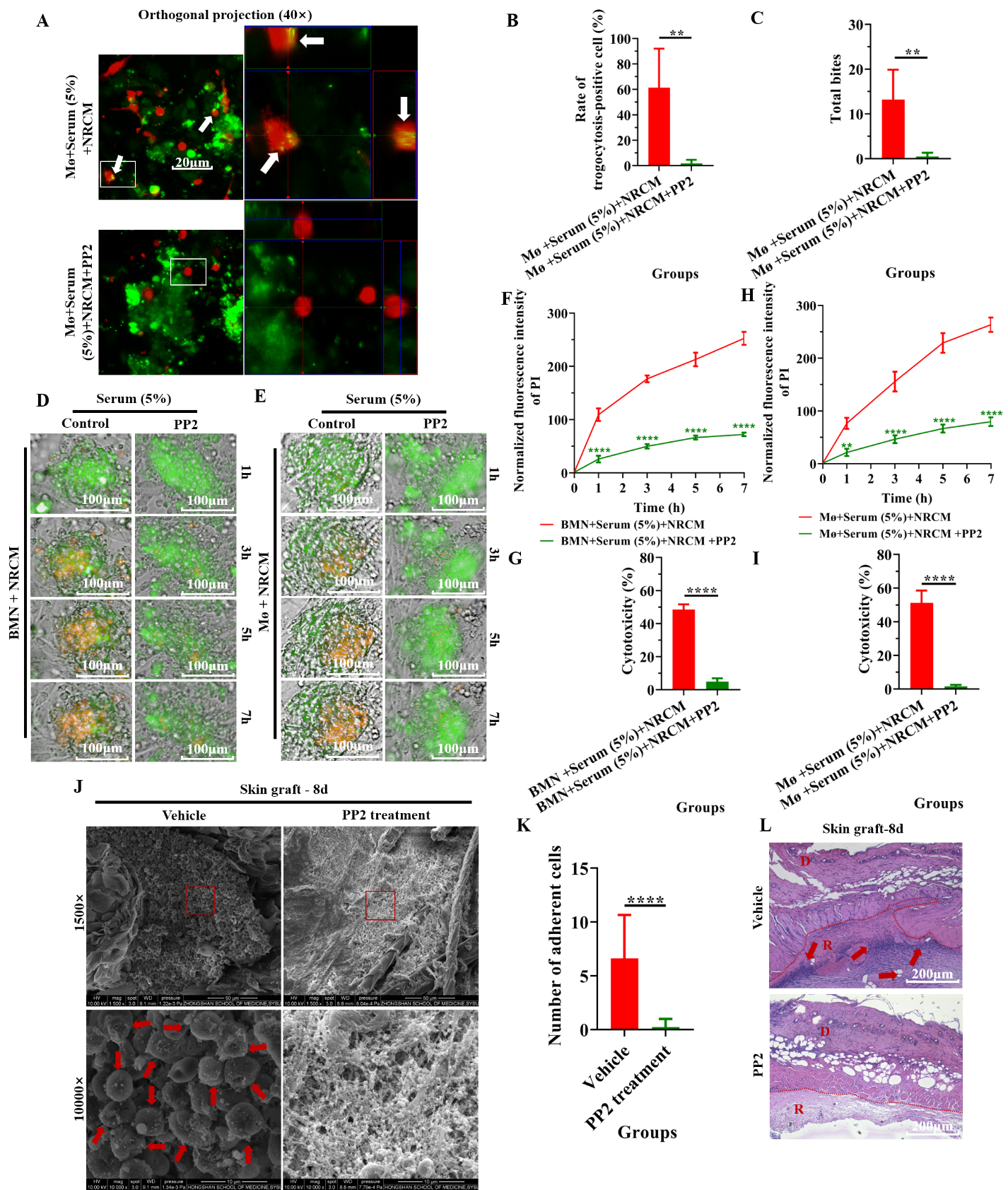
**

**Fig. S4. PP2 inhibits trogocytosis of C57BL/6-derived BMN/Mø to NRCM.**

**A** Representative images showing Mø (red) of C57 mice trogocytosing the NRCM (green) after co-cultured for 12h in the presence of PP2 *in vitro*. The white box contains a partially enlarged view. The white arrows indicate Mø with positive trogocytosis. Scale bar = 20µm. Magnification = 400×. Three C57BL/6 mice were used and repeated thrice with similar results. **B and C** Quantitative analysis of trogocytosis-positive Mø (B) and total bites of Mø (C) from A. Data are expressed as mean ± SD (n = at least 3 per group) and repeated twice with similar results. Statistical analysis was performed using Student’s t test. *P < 0.05, **P < 0.01, ***P < 0.001, ****P < 0.0001. Scale bar = 20µm. Magnification = 400×. **D, E** Representative images showing the apoptosis of NRCM (orange) at different times after co-culture with BMN (D) / Mø (E) of C57 mouse in the presence of PP2 after co-cultured for 7h. NRCM were labeled with CFDA SE (green) and the apoptosis of NRCM was detected by propidium iodide staining (orange). Scale bar =100µm. Magnification =20×. Three C57BL/6 mice were used and repeated thrice with similar results. **F-I** Determination of the damage to NRCM induced by C57 mouse BMN/Mø in the presence of PP2 *in vitro* after co-cultured for 7h by propidium iodide staining (orange) (F and H) and after co-cultured for 6h by non-radioactive cytotoxicity assay (Released LDH in culture supernatants) (G and I). Data are expressed as mean ± SD (n = 3 per group) and repeated thrice with similar results. Statistical analysis was performed using Student’s t test. *P < 0.05, **P < 0.01, ***P < 0.001, ****P < 0.0001. **J** Representative SEM images showed cell adhesion (as indicated by the red arrow) to the recipient contact surface of the grafts in control mice (vehicle) and PP2-treated recipient mice at 8 days post-transplantation. Scale bar =50µm/10µm. Magnification = 1500×/10000×. n= 5 mice per group. **K** Quantitative analysis of the number of adherent cells. The numerical value represents the number of cells in the field of view at a magnification of 10000. Data are expressed as the mean ± SD. Statistical analysis was performed using Student’s t test. **P* < 0.05, ***P* < 0.01, ****P* < 0.001, *****P* < 0.0001. n= 5 mice per group. **L** Representative H&E staining images of Balb/c mouse skin grafts in control mice (vehicle) and PP2-treated recipient mice at 8 days post-transplantation. Arrows indicate the aggregation of inflammatory cells. D: donor; R: recipient. Scale bar =200µm. Magnification = 100×. n= 5 mice per group.


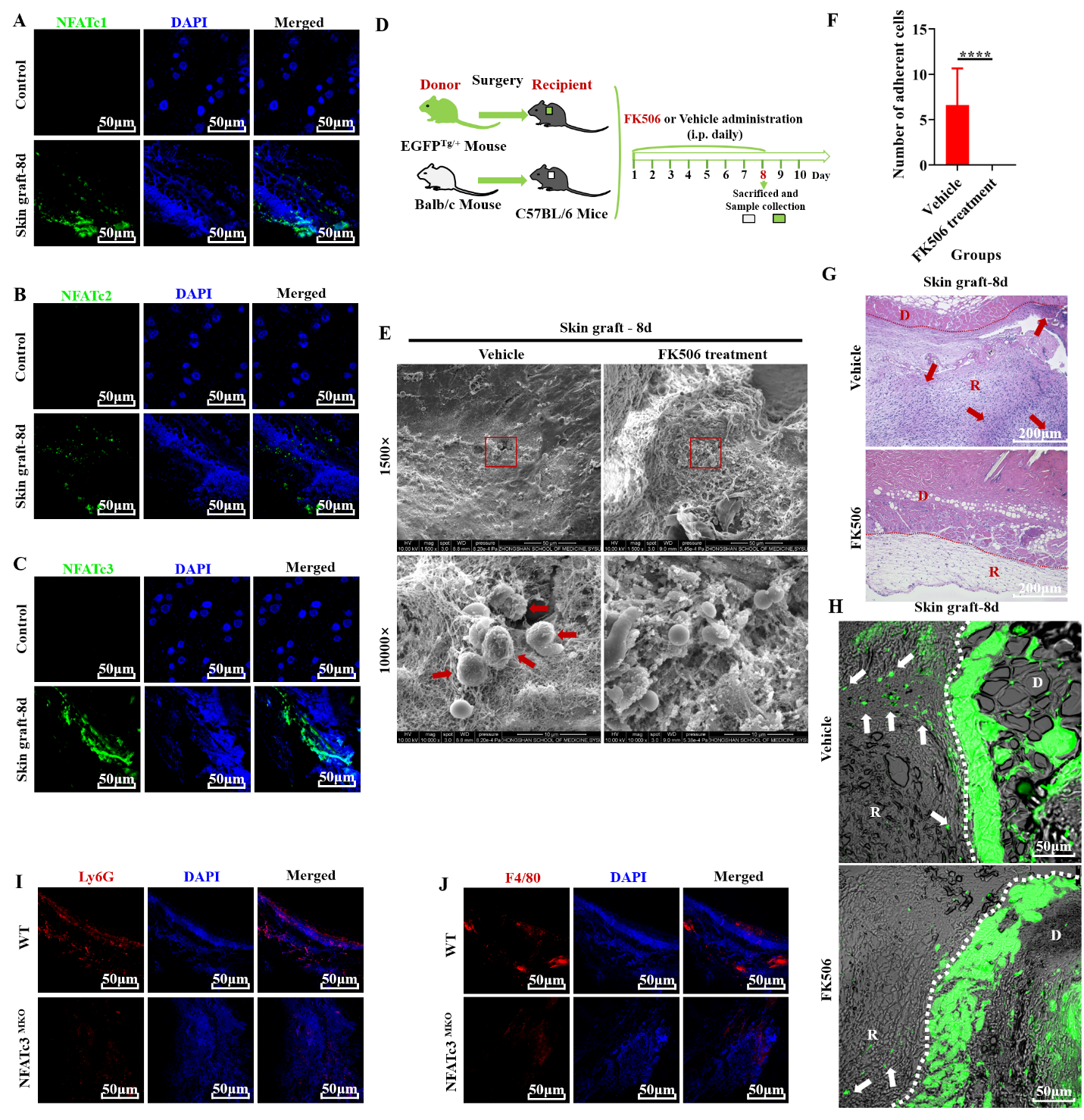


**Fig. S5. NFATc3 regulated BMN/Mø-mediated trogocytosis in skin graft models.**

**A-C** Immunofluorescence detecting the expression of NFATc1, NFATc2 and NFATc3 (green) in 8-day skin grafts and normal recipient skin (control). Scale bar = 50µm. Magnification = 200×. n= 5 mice per group. **D** Schematic illustration of FK506 administration after skin graft from EGFP^Tg/+^ mouse or Balb/c mouse into wild-type C57BL/6 mice. **E** Representative SEM images showed cell adhesion (as indicated by the red arrow) to the recipient contact surface of the grafts in control mice (vehicle) and FK506-treated mice as recipients at 8 days post-transplantation (n = at least 3 per group). Scale bar =50µm/10µm. Magnification = 1500×/10000×. n= 5 mice per group. **F** Quantitative analysis of the number of adherent cells. The numerical value represents the number of cells in the field of view at a magnification of 10000. Data are expressed as the mean ± SD. Statistical analysis was performed using Student’s t test. **P* < 0.05, ***P* < 0.01, ****P* < 0.001, *****P* < 0.0001. n= 5 mice per group. **G** Representative H&E staining images of Balb/c mouse skin grafts in control mice (vehicle) and FK506-treated mice as recipients at 8 days post-transplantation. Arrows indicate the aggregation of inflammatory cells. D: donor; R: recipient. Scale bar =200µm. Magnification = 100×. n= 5 mice per group. **H** Representative images showed trogocytosis on the interface between donor skin (green; D) and recipient skin (TL-PH; R) in control mice (vehicle) and FK506-treated recipient mice at 8 days post-transplantation. The white arrows indicate trogocytosing cells. Scale bar = 50µm. Magnification = 200×. n= 5 mice per group. **I, J** Representative images of immunofluorescence staining of Ly6G (I, the marker of neutrophils, red) or F4/80 (J, the marker of macrophages, red) in the recipient tissue attached to skin grafts. Nuclei are stained with DAPI (blue). WT: wild-type C57BL/6 mice as transplant recipients; NFATc3^MKO^: NFATc3^MKO^ mice as transplant recipients. Scale bar = 50µm. Magnification = 200×. n= 5 mice per group.

**
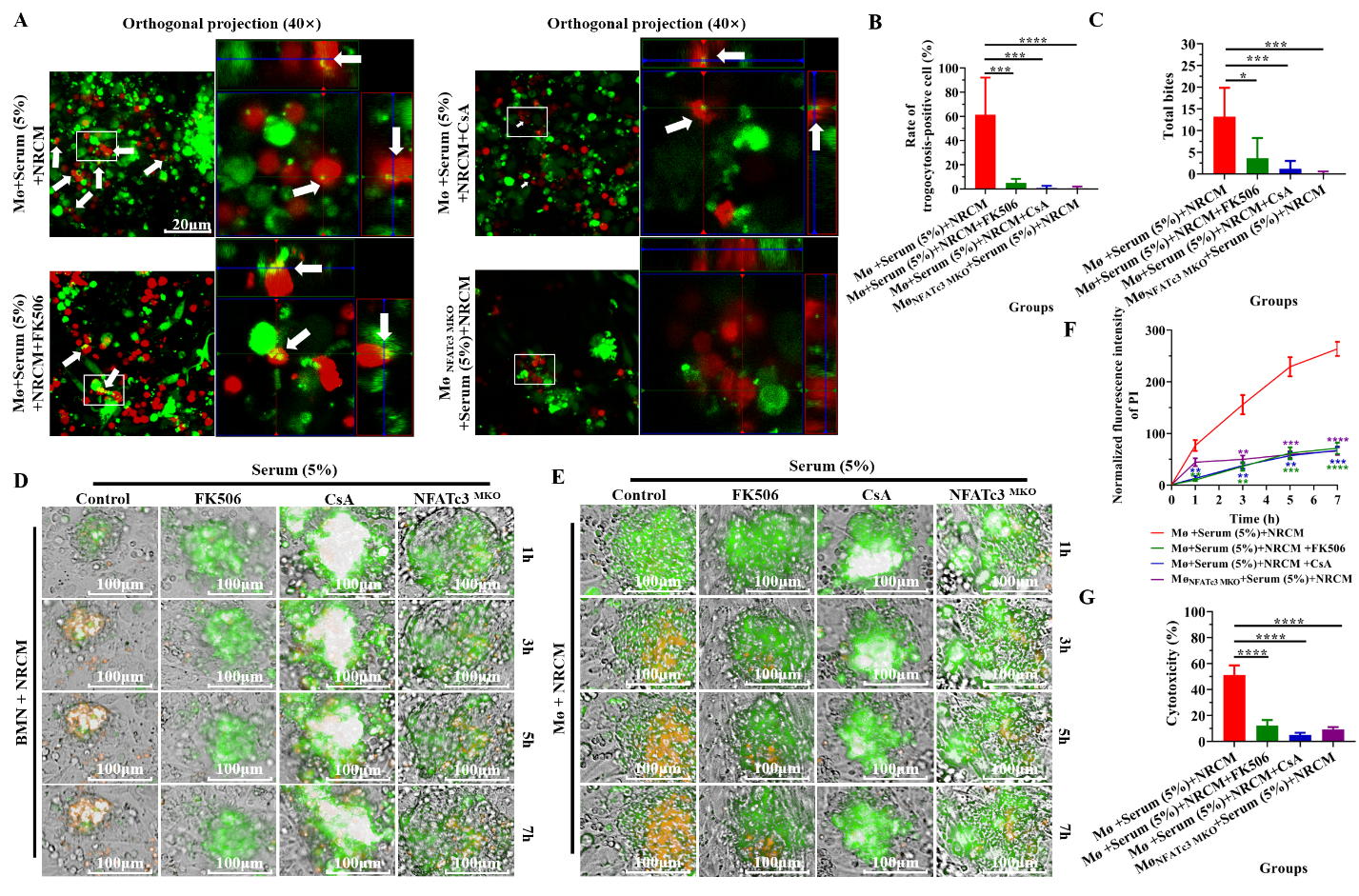
Fig. S6. NFATc3 regulated BMN/Mø-mediated trogocytosis in the mimicking system.**

**A-C** Analysing Mø-mediated trogocytosis after blocking NFAT activation with FK506, CsA and NFATc3^MKO^ Mø *in vitro*. The white arrows indicate trogocytosing cells. Scale bar = 20µm. Magnification = 400×. Data are expressed as mean ± SD (n = at least 3 per group) and repeated twice with similar results. Statistical analysis was performed using one-way ANOVA with Dunnett's multiple comparisons test. *P < 0.05, **P < 0.01, ***P < 0.001, ****P < 0.0001. Three C57BL/6 mice were used and repeated thrice with similar results. **D, E** Representative images showing NRCM damage mediated by C57 mouse BMN (D) / Mø (E) (Bright field) with NFAT inhibitors or NFATc3^MKO^ cells at different time points after co-cultured for 7h. NRCM were labeled with CFDA SE (green) and the apoptosis of NRCM was indicated by propidium iodide staining (orange) *in vitro*. Scale bar =100µm. Magnification =20×. Three C57BL/6 mice were used and repeated thrice with similar results. **F, G** Quantitative analysis of the damage to NRCM determined by PI staining (F) and LDH concentrations in culture supernatants (G) after blocking NFAT activation with FK506, CsA and NFATc3^MKO^ Mø *in vitro*. Scale bar =100µm. Magnification =20×. Data are expressed as mean ± SD (n = at least 3 per group) and repeated twice with similar results. Statistical analysis was performed using one-way ANOVA with Dunnett's multiple comparisons test. *P < 0.05, **P < 0.01, ***P < 0.001, ****P < 0.0001.


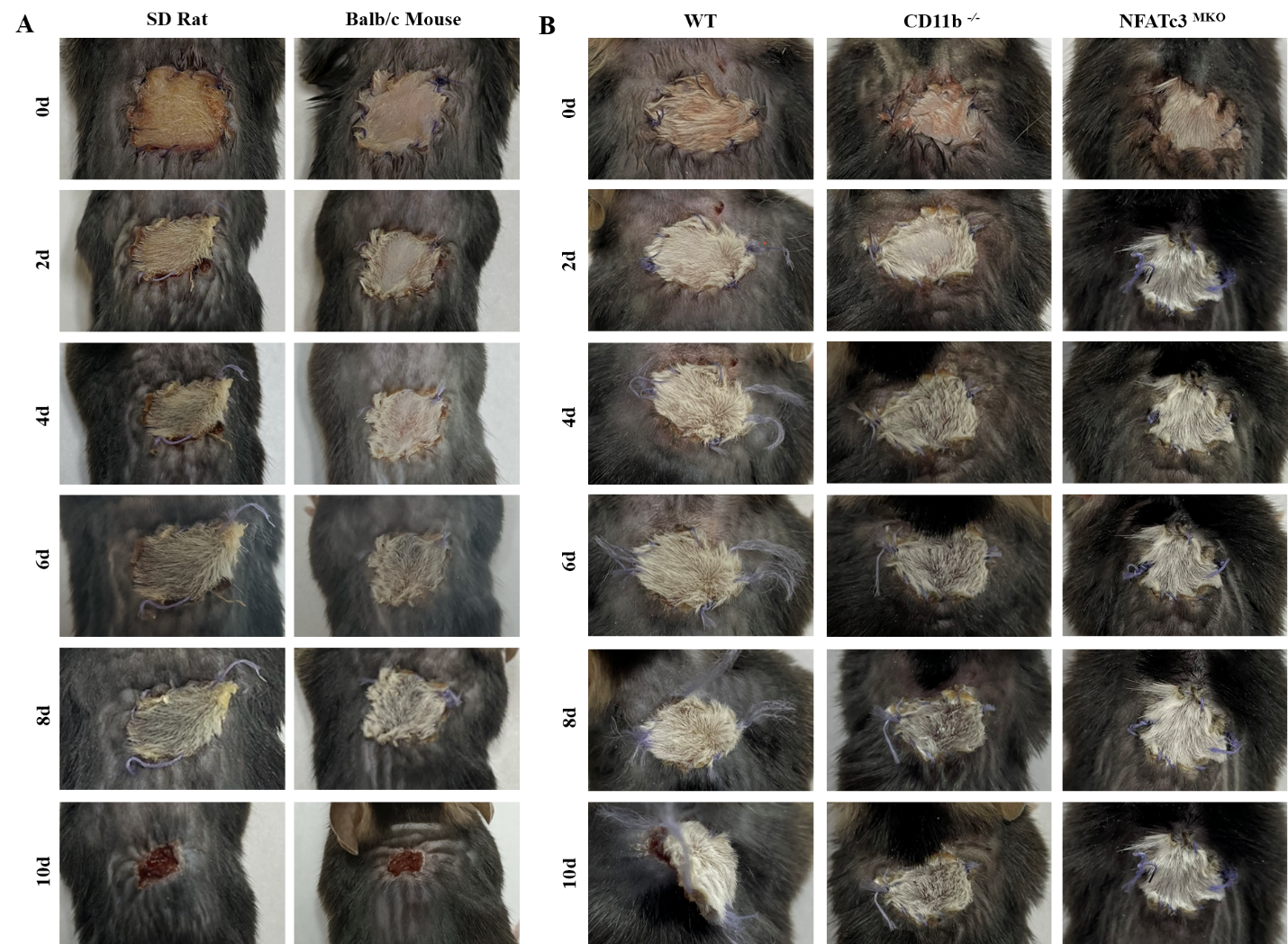


**Fig. S8.** **Changes in grafts at different time points after transplantation.**

**A** Representative images showing skin from SD Rat or Balb/C mouse was transplanted to the nape of wild-type C57 mice. **B** Representative images showing skin from Balb/C mouse was transplanted to the nape of wild-type C57 mice, CD11b^-/-^ mice or NFATc3^MKO^ mice.


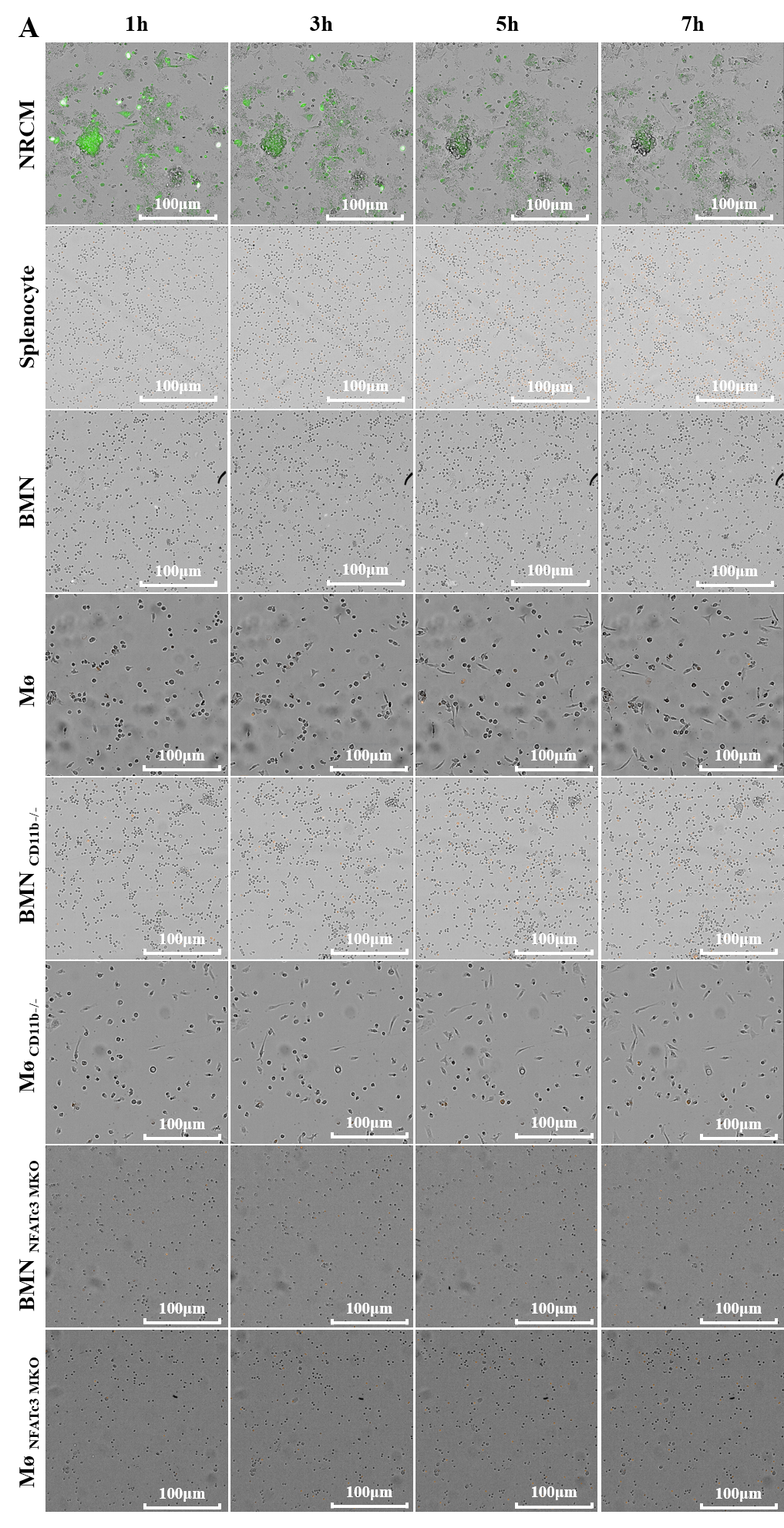


**Fig. S9. The apoptosis of vehicle-only cells controls in co-culture system changed at different times.**

**A** Representative images showing the apoptosis (orange) at different times of NRCM, splenocytes, BMN, Mø, BMN _CD11b-/-_, Mø _CD11b-/-_, BMN _NFATc3 MKO_, Mø _NFATc3 MKO_ (bright field) from C57BL/6 mouse, CD11b^-/-^ mouse or NFATc3 ^MKO^ mouse. The NRCM were labeled with CFDA SE (green) and the apoptosis of NRCM was detected by propidium iodide staining (orange). Scale bar =100µm. Magnification =20×.

**Table S1. Quantitative Real Time qPCR Primer sequences**

| Gene | Forward (5’-3’) | Reverse (5’-3’) |
| --- | --- | --- |
| *GAPDH* (Mouse)  *NFATc1* (Mouse) | ACTCCACTCACGGCAAATTC  GGAGAGTCCGAGAATCGAGAT | TCTCCATGGTGGTGAAGACA  TTGCAGCTAGGAAGTACGTCT |
| *NFATc2* (Mouse)  *NFATc3* (Mouse)  *NFATc4* (Mouse) | GCAGCAGATTTGGGAGATGG  GCTCGACTTCAAACTCGTCTT  GCTACAGCCAGCTATGAAGC | TGGCTGACTTCGTTTCCTCT  GATGTGGTAAGCCAAGGGATG  GCTTCAGGATTCCAGCACAG |
